# Distinctive Patterns of Flavonoid Biosynthesis in Roots and Nodules of *Datisca glomerata* and *Medicago spp.* Revealed by Metabolomic and Gene Expression Profiles

**DOI:** 10.3389/fpls.2018.01463

**Published:** 2018-10-10

**Authors:** Isaac Gifford, Kai Battenberg, Arpana Vaniya, Alex Wilson, Li Tian, Oliver Fiehn, Alison M. Berry

**Affiliations:** ^1^Department of Plant Sciences, University of California, Davis, Davis, CA, United States; ^2^West Coast Metabolomics Center, University of California, Davis, Davis, CA, United States

**Keywords:** flavonoid, root nodule, symbiosis, actinorhizal, legume, metabolome profile, gene expression profile, phenylpropanoid

## Abstract

Plants within the Nitrogen-fixing Clade (NFC) of Angiosperms form root nodule symbioses with nitrogen-fixing bacteria. Actinorhizal plants (in Cucurbitales, Fagales, Rosales) form symbioses with the actinobacteria *Frankia* while legumes (Fabales) form symbioses with proteobacterial rhizobia. Flavonoids, secondary metabolites of the phenylpropanoid pathway, have been shown to play major roles in legume root nodule symbioses: as signal molecules that in turn trigger rhizobial nodulation initiation signals and acting as polar auxin transport inhibitors, enabling a key step in nodule organogenesis. To explore a potentially broader role for flavonoids in root nodule symbioses across the NFC, we combined metabolomic and transcriptomic analyses of roots and nodules of the actinorhizal host *Datisca glomerata* and legumes of the genus *Medicago.* Patterns of biosynthetic pathways were inferred from flavonoid metabolite profiles and phenylpropanoid gene expression patterns in the two hosts to identify similarities and differences. Similar classes of flavonoids were represented in both hosts, and an increase in flavonoids generally in the nodules was observed, with differences in flavonoids prominent in each host. While both hosts produced derivatives of naringenin, the metabolite profile in *D. glomerata* indicated an emphasis on the pinocembrin biosynthetic pathway, and an abundance of flavonols with potential roles in symbiosis. Additionally, the gene expression profile indicated a decrease in expression in the lignin/monolignol pathway. In *Medicago sativa*, by contrast, isoflavonoids were highly abundant featuring more diverse and derived isoflavonoids than *D. glomerata.* Gene expression patterns supported these differences in metabolic pathways, especially evident in a difference in expression of cinnamic acid 4-hydroxylase (C4H), which was expressed at substantially lower levels in *D. glomerata* than in a *Medicago truncatula* transcriptome where it was highly expressed. C4H is a major rate-limiting step in phenylpropanoid biosynthesis that separates the pinocembrin pathway from the lignin/monolignol and naringenin-based flavonoid branches. Shikimate *O*-hydroxycinnamoyltransferase, the link between flavonoid biosynthesis and the lignin/monolignol pathway, was also expressed at much lower levels in *D. glomerata* than in *M. truncatula*. Our results indicate (a) a likely major role for flavonoids in actinorhizal nodules, and (b) differences in metabolic flux in flavonoid and phenylpropanoid biosynthesis between the different hosts in symbiosis.

## Introduction

Root nodule symbioses (RNS) develop as symbiotic associations between nitrogen-fixing bacteria and certain host plants, resulting in the formation of the root nodule, a specialized organ for nitrogen fixation and assimilation. The root nodule provides a number of functions in RNS, primarily serving as a site for the exchange of carbon and energy-containing molecules from the host for nitrogen-containing molecules from the microsymbiont, and also as an environment to help regulate oxygen concentration to protect the nitrogenase enzyme complex. The bacteria capable of establishing these symbioses fall into two distantly related groups: the proteobacterial rhizobia and the actinobacterial genus *Frankia*. The host plants, on the other hand, all belong to a single clade of angiosperms known as the Nitrogen-fixing Clade (NFC) (Soltis et al., 1995), consisting of the order Fabales (nodulated by rhizobia) and three orders that include the actinorhizal plants, Cucurbitales, Fagales, and Rosales (nodulated by *Frankia*).

The establishment of RNS involves a signal–mediated recognition interaction between host and microsymbiont within the rhizosphere, followed by the entry of the microsymbiont into root cells, and ultimately by nodule organogenesis (Gage, 2004). Early stages of organogenesis involve the division of cortical cells and cell expansion during invasion by the microsymbiont, followed by nodule organogenesis and maturation of nitrogen-fixing symbiotic tissue. During the maturation phase, cells within the developing nodule undergo endoreduplication, increasing in volume and becoming more transcriptionally active to promote symbiotic interactions (Vinardell et al., 2003). In both the legume and actinorhizal symbioses several of the initial steps in the internal signaling pathway leading to nodule establishment are conserved. Initial signaling interactions are activated via the Common Symbiotic Pathway, a set of genes shared with the more ancient arbuscular mycorrhizal symbioses (Oldroyd, 2013), indicating a shared evolutionary origin within the NFC (Markmann and Parniske, 2009; Battenberg et al., 2018), followed by a RNS-specific gene expression cascade (Oldroyd, 2013).

**FIGURE 1:**
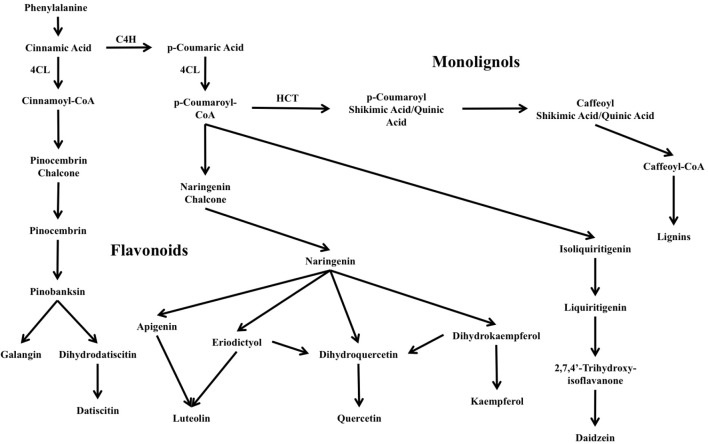
Overview of major flavonoid biosynthesis pathways in *Datisca glomerata*. Also shown is the lignin/monolignol pathway (upper-right). C4H: cinnamic acid 4-hydroxylase. 4CL: 4-coumarate CoA-ligase. HCT: shikimate *O*-hydroxycinnamoyltransferase.

Flavonoids are ubiquitous secondary metabolites synthesized by the phenylpropanoid pathway. The flavonoid pathway is one of two major branches in plant phenylpropanoids, the other being monolignol/lignin biosynthesis, and is responsible for producing a wide range of metabolites fundamental for plant structure and function and plant–organism interactions including symbiotic signaling in RNS and nodule organogenesis and development. Plant flavonoids are also key molecules in pigmentation and signaling for pollinator attraction, herbivore or pathogen deterrence, reduction of damage from reactive oxygen species, UV light protection, and regulation of development (Shirley, 1996; Buer et al., 2010). The reactions linking the flavonoid and monolignol/lignin branches of the pathway and the interconversions among flavonoid classes are illustrated in **Figure 1**.

In the establishment of root nodule symbiosis in many legume genera, flavonoids produced by the host are recognized by rhizobia in the rhizosphere, primarily through the receptor-transcription factor NodD. This, in turn, triggers the expression of the other *nod* genes (*nodA, nodB, nodC*), which synthesize a lipochitooligosaccharide molecule, the Nod factor, which is secreted by the rhizobia (Long, 1996), that in turn triggers host cellular responses leading to root-nodule development (Oldroyd, 2013). A wide range of flavonoid molecules, both aglycones and glycosides, has been identified as nodulation signals in legume symbioses (Peck et al., 2006). To date, no molecule similar to Nod factor has been identified in the *Frankia*-actinorhizal symbioses; however, genomes of some members of the Cluster 2 group of *Frankia* contain homologs of the rhizobial *nodABC* genes that are expressed during symbiosis (Persson et al., 2015; Nguyen et al., 2016), suggesting that a Nod factor may play a role in at least some actinorhizal symbioses. Cluster 2 *Frankia* genomes do not contain any identified homologs of *nodD*, leaving the mechanism of induction of transcription unknown, and the role of flavonoids in actinorhizal-*Frankia* signaling to be determined.

After the initial signaling steps in nodulation, flavonoids play a continuing role in legume nodule development. Flavonoids are known to bind to and inhibit auxin transporters, leading to a disruption of polar auxin transport (Wasson et al., 2006). The accumulation of auxin within certain cortical cells in the root triggers cell division and proliferation, which, in turn, leads to the localized induction of the nodule (Mathesius et al., 1998). Recent studies have suggested that the production of flavonoids during nodule organogenesis is itself a response to increased cytokinin production following the perception of the symbiotic Nod factor (Mathesius et al., 1998). Similar effects in the actinorhizal symbioses have been far less studied; however, it has been shown that inhibition of auxin gradients with an auxin influx inhibitor in the actinorhizal host *Casuarina glauca* led to decreased nodulation, suggesting a similar role for auxin in actinorhizal symbioses (Péret et al., 2007; Champion et al., 2015). Additionally, flavonoids have been suggested to play a role in triggering endoreduplication through DNA breaks resulting in anaphase arrest (Cantero et al., 2006).

In this study, flavonoids from the metabolomes of roots and nodules of the actinorhizal host *Datisca glomerata* were compared with those of the legume *Medicago sativa*. Additionally, the metabolome results were compared with available transcriptomes of *D. glomerata* and *Medicago truncatula* (Roux et al., 2014; Battenberg et al., 2018). Both hosts were found to synthesize phenylpropanoid derivatives of the flavonoid branch in several different categories including flavones, flavanones, and isoflavonoids, but with different apparent patterns of metabolic flux.

## Materials and Methods

### Growth Conditions and Nodule Sampling

*Datisca glomerata* seeds were collected from wild plants growing in Gates Canyon, Vacaville, CA, United States, germinated, grown, and inoculated in a greenhouse at University of California, Davis, under conditions as described in Battenberg et al. (2018). The seedlings were inoculated with crushed *Ceanothus thyrsiflorus* nodules containing *Frankia* originally sampled in Sagehen Experimental Forest (Truckee, CA, United States). Until inoculation, one-half-strength Hoagland’s solution with nitrogen (Hoagland and Arnon, 1950) was applied weekly. After inoculation, one-half-strength Hoagland’s solution without nitrogen was applied weekly.

Uninoculated roots, inoculated roots, and root nodules were collected for analysis from four individual plants per treatment. Inoculated roots and nodules were collected from the same plants; both were harvested 100 days after inoculation. Uninoculated roots were collected from the same plants sampled for inoculated roots and nodules, prior to inoculation. Sampling methods are described in detail in Battenberg et al. (2018). Collected samples were flash frozen in liquid nitrogen and stored at -80°C until use. For detailed information on samples collected, see **Supplementary Table S1**. Root and nodule samples from individual plants were ground in liquid nitrogen in a mortar and pestle, prior to extraction.

Mature individual *M. sativa* plants were collected from field plantings at the Russell Ranch Sustainable Agriculture Facility, University of California, Davis, and maintained in a greenhouse at University of California, Davis. Roots and root nodules were collected and sampled from four individual plants per treatment, as described above.

### Flavonoid Extraction

Root nodules and inoculated roots of *M. sativa* and *Datisca glomerata* were extracted using 80:20 MeOH/H_2_O. 40 mg of samples were extracted with 2000 μL of cold solvent. Samples were then mixed for 10 s using Mini Vortexer (VWR, Radnor, PA, United States). Samples were then centrifuged for 5 min at 14,000 relative centrifugal force (RCF) using an Eppendorf Centrifuge 5415D (Hauppaugee, NY, United States). After removing the supernatant, samples were dried using a Labconco CentriVap Concentrator (Kansas City, MO, United States). Dried samples were resuspended in 110 μL of 10:90 ACN/H_2_O with 1 μg/mL 12-(cyclohexylcarbamoylamino)dodecanoic acid (CUDA) for LC-MS/MS analysis.

### Metabolomic Analysis

For metabolomic analysis of flavonoids and related molecules, a comparison was made between root nodules and inoculated roots collected from the same plants. Chromatography was performed using a Thermo Vanquish UHPLC instrument, a Phenomenex Kinetex C18 column (100 × 2.1 mm, 1.7 μm) with a KrudKatcher Ultra HPLC in-line filter (0.5 μm Depth Filter × 0.004 in ID). The mobile phases were H_2_O with 0.1% acetic acid (A) and ACN with 0.1% acetic acid (B). Gradient elution was performed at a flow rate of 0.5 mL/min under the following program: from 0 to 10 min B changed linearly from 10 to 90%, held at 90% B for 2.50 min, returned to 10% B over the next 2.50 min, and held at 10% B for equilibration over 5 min. The column temperature was kept at 45°C. The LC method was modified from Ma et al. (2016). MS/MS data were acquired on a high resolution Thermo Q Exactive HF mass spectrometer in positive electrospray ionization (ESI) mode under the following operating parameters: sheath gas flow rate at 60, auxiliary gas flow rate at 25, sweep gas flow rate at 2, spray voltage at 3.60 kV, capillary temperature at 300°C, S-lens RF level at 50, and auxiliary gas heater temperature at 370°C. Mass spectral data were collected using full scan MS1 and data-dependent MS/MS. Full scan MS1 had the following parameters: scan range from a *m/z* 150–2000 with the resolution set to 120,000, AGC target set to 1 × 10^6^, and maximum ion injection set to 300 ms. Data-dependent MS2 had the following parameters; scan range from *m/z* 150–2000 with the resolution set to 15,000, AGC target set to 1 × 10^5^, maximum injection time set to 50 ms, loop count set to 3, and TopN set to trigger the top-3 most abundant ions, with an isolation window of 1.0 *m/z*, and Higher Energy Collisional Dissociation (HCD) was conducted using three normalized collision energies; 35, 45, and 65%. The observed MS/MS spectra have an HCD collision energy of 48.33%, spectra from the three normalized collision energies are automatically averaged. The injection volume for each sample was 2 μL.

In metabolomics compounds are routinely identified by data processing tools which match MS/MS spectra against mass spectral reference libraries and use cheminformatics to provide spectral interpretation (Neumann and Bocker, 2010; Dunn et al., 2012; Cajka and Fiehn, 2015). Here we have used MS-DIAL software version 2.82 (Tsugawa et al., 2015) was used to process the raw data and metabolites were reported using a 0% peak count filter to keep all detected features. MS-DIAL was used for data deconvolution, peak alignment, and compound identification by searching mass spectral refrence libraries. Compound identifications were made based on an in-house accurate mass and retention time (m/z-RT) library created from the QC reference standard mix and the following tandem mass spectral libraries; MassBank, ReSpect, MetaboBASE, HMDB, GNPS, NIST 17 MS/MS, FAHFA, LipidBlast, and iTree MS/MS only. The tandem mass spectral libraries were downloaded in an msp format from MassBank of North America (MoNA) which was later used in MS-DIAL. MS-FLO (Mass Spectral Feature List Optimizer) was used as post processing tool to optimize the feature list from MS-DIAL to remove duplicate and isotopic features and identifiy ions adduct (DeFelice et al., 2017).

After reduction, annotations were also labeled with Metabolomics Standards Intitaive (MSI) levels and mass error (mDa) to provide confidence in each annotation. Level 1 is the highest level of identification. It is described as using two or more orthogonal data from an authentic standard. Level 2 is when only one set of reference data from an authentic standard has been used, for example, either using an in-house accurate m/z-RT library or using mass spectral library search for MS/MS matching. Level 3, is similar to Level 2 where a match can be made with either a m/z-RT library or a MS2 library, but the match lacks high accuracy. Lastly, Level 4 indicates those metabolites that are unknown (Sumner et al., 2007; Schymanski et al., 2014). The observed MS spectra of identified flavonoid compounds in *D. glomerata* and *M. sativa* are shown in **Supplementary Figures S1**, **S2**, respectively, as head-to-tail comparisons of experimental and reference MS/MS spectra.

### Metabolome and Phenylpropanoid Pathway Analysis

Flavonoid molecules detected by LCMS were annotated by their International Chemical Identifier (InChIKey) and separated into subclasses of flavonoids, isoflavonoids, flavones, flavonols, and anthocyanins by ClassyFire (Feunang et al., 2016). For each plant, the average peak height of flavonoids in each plant was calculated and molecules with average peak heights above the mean were considered highly abundant. Significant differences between roots and nodules were identified with two-tailed Welch’s *t*-tests. A comparison of the overall proportions of flavonoids annotated by class in the metabolomes of *D. glomerata* and *M. sativa* was performed with a chi-square test. T-tests and chi-square tests were performed in R using a significance level of *p* < 0.05.

For molecules with multiple annotated isotopes, only the dominant isotope was used, identified by the highest peak height across all samples. Phenylpropanoid biosynthesis pathways were obtained from KEGG (Kanehisa et al., 2016). Maps used included: Flavonoid Biosynthesis, Flavone and Flavonol Biosynthesis, Anthocyanin Biosynthesis, Isoflavonoid Biosynthesis, and Phenylpropanoid Biosynthesis. Within each class molecules that were significantly different between root and nodule LC-MS samples were grouped by their structural similarity using molecular structures acquired from PubChem (Kim et al., 2016) to reference molecules that included: eriodictyol, naringenin, liquiritigenin, daidzein, genistein, glycitein, formononetin, kaempferol quercetin, luteolin, apigenin, cyanidin, pinocembrin, pinobanksin, galangin, and chrysin. In cases where an enzyme for synthesizing a particular molecule could not be identified, putative pathways were inferred, placing molecules together in groups if their chemical structures shared diagnostic structures of the reference molecules including: a 2C–3C carbon double bond, a 3 carbon hydroxyl, 3′ or 4′ hydroxyls, or a 2 or 3 carbon benzene ring.

### Abundant Compound Verification by HPLC, UV Absorption and LC-MS

To investigate the effect of *Frankia* interactions with *D. glomerata* on flavonoid production uninoculated roots, roots inoculated with *Frankia*, and nodules of *D. glomerata* were collected for a second analysis of selected abundant flavonoids. The ground tissue (100 mg) was extracted in 300 μL of 80% methanol, with incubation in an ultrasonic water bath for 20 min at 30°C. The extract was then centrifuged twice for 10 min each at 17,000 × *g*. The supernatant was transferred to an HPLC vial; 10 μL of the supernatant was injected on a reverse phase HPLC and analyzed as previously described (Knollenberg et al., 2018). Major metabolites (i.e., abundant HPLC peaks) that exhibited differential accumulation among roots and nodules were collected and analyzed by mass spectrometry (MS) in negative mode and MS/MS using an established method (Ono et al., 2016). The flavonoid metabolites were tentatively identified based on their retention times, UV absorption spectra, as well as MS and MS/MS data, taking into consideration phenolic metabolites previously reported to accumulate in roots of Datiscaceae (Bohm, 1988). In addition, authentic standards of kaempferol, luteolin, and genistein (Sigma Aldrich, St. Louis, MO, United States) were analyzed in parallel with the *D. glomerata*.

One-way analysis of variation (ANOVA) followed by Tukey’s HSD test were performed on the metabolite data using JMP (SAS Institute, Cary, NC, United States).

### Transcriptome Analysis of *D. glomerata* and *M. truncatula* Phenylpropanoid Pathways

Transcript annotations from Battenberg et al. (2018) were used for *D. glomerata* and *M. truncatula* (Roux et al., 2014). The transcriptome of *M. truncatula* was chosen because of its depth of coverage and comparable stage of nodulation. Enzyme Commission (EC) number annotations were made with InterProScan v5.21 (Jones et al., 2014) and Trinotate v3.0.1 (Haas et al., 2013). Transcripts annotated with Enzyme Commission (EC) numbers belonging to the KEGG phenylpropanoid biosynthetic pathways listed above were identified in each transcriptome. Expression fold changes between nodules and roots (Log-scale) were used to generate heat maps in Microsoft Excel. To determine genes important for symbiosis and compare relative expression levels of genes between nodules of the two hosts, transcripts in the 90th percentile or above ranked by transcripts per million (TPM) in the full transcriptomes were considered “highly expressed,” and transcripts below the 50th percentile were considered “low expression.” Statistical significance of expression level differences between *D. glomerata* and *M. truncatula* nodules were determined with one-sample *t*-tests comparing the percentile ranks of the most highly expressed transcript for each gene in the *D. glomerata* transcriptome with the most highly expressed transcript from *M. truncatula* (*p* < 0.05).

## Results

### Metabolomics Analysis Revealed Abundant and Diverse Flavonoid Accumulation in *D. glomerata* and *M. sativa*

**FIGURE 2:**
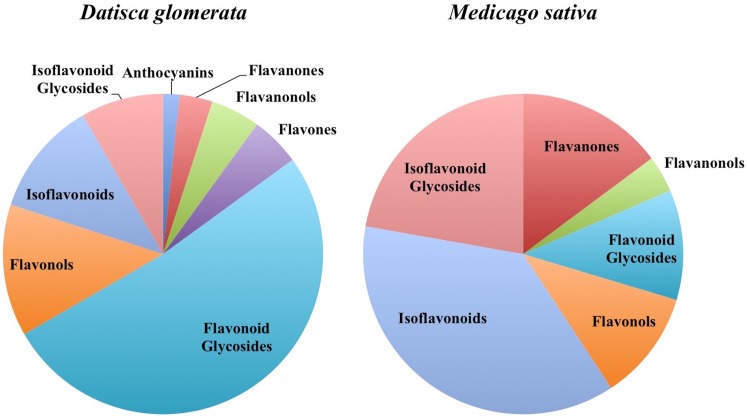
Distribution of flavonoids in *D. glomerata* and *M. sativa* roots and nodules by class.

**FIGURE 3:**
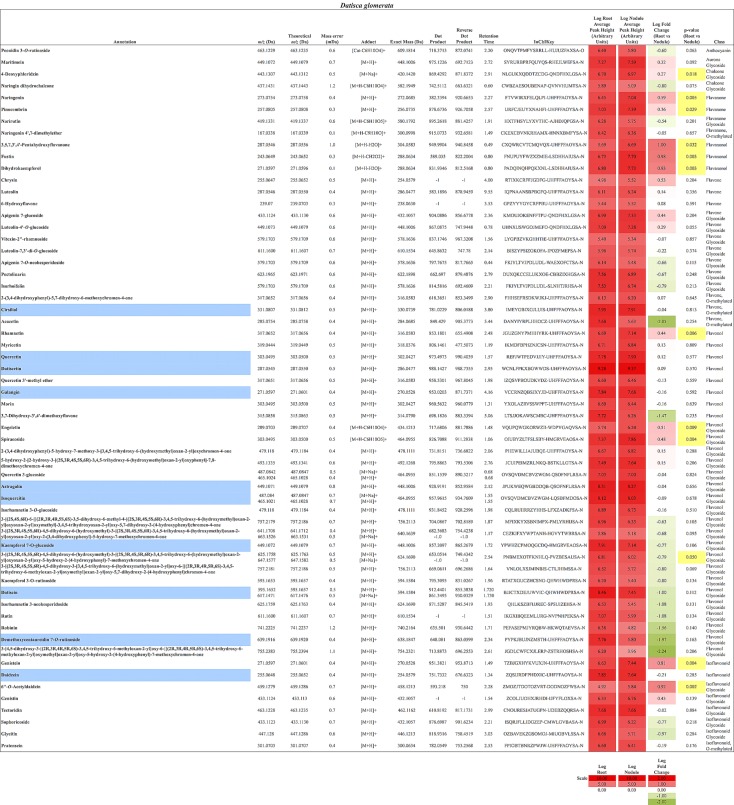
Flavonoids identified in *D. glomerata* roots and nodules. All annotations conform to MSI level 2. For each flavonoid the abundance in both the roots and nodules is given as the log average peak height of 10 samples and presented as a heat-map. The names of molecules considered highly abundant are highlighted in blue. Significant changes in abundance between roots and nodules are highlighted in yellow.

**FIGURE 4:**
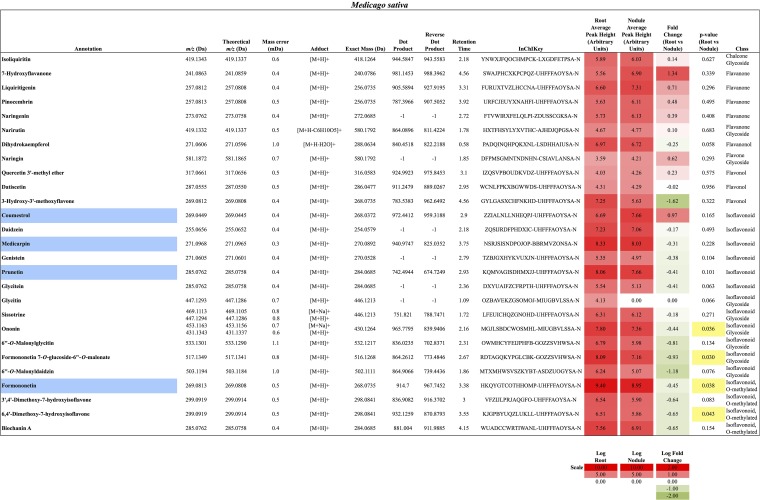
Flavonoids identified in *M. sativa* roots and nodules. All annotations conform to MSI level 2. For each flavonoid the abundance in both the roots and nodules is given as the log average peak height of four samples and presented as a heat-map. The names of molecules considered highly abundant are highlighted in blue. Significant changes in abundance between roots and nodules are highlighted in yellow.

In total, 384 compounds from *D. glomerata* roots and nodules were initially annotated as flavonoids, however, only 60 metabolites were matched against spectra from an MS/MS library at high levels of identification (MSI Level 1, 2, or 3) and the rest were reclassified as unknowns (**Supplementary Table S2**). The relative distributions of annotated flavonoids by class in *D. glomerata* and *M. sativa* are presented in **Figure 2** and listed in **Figures 3**, **4** for *D. glomerata* and *M. sativa*, respectively. Of the 60 annotated flavonoids in *D. glomerata* 24 were aglycones and 36 were glycosides, the majority of which were flavonols (**Figure 3**). In *M. sativa* 281 compounds were initially annotated as flavonoids but only 27 compounds met the MSI Level 3 or better, of which nine were glycosylated. *M. sativa* root and nodule flavonoids were predominantly isoflavonoids (**Figure 4**). These differences in flavonoid distribution between *D. glomerata* and *M. sativa* were strongly significant (*p* < 5 × 10^-8^, **Supplementary Table S3**). With the exception of glycitin in *M. sativa* nodules all flavonoids identified were detected in both the roots and nodules of their respective plant (**Figures 3**, **4**).

Ten flavonoids were highly abundant in nodules of *D. glomerata*. Abundant aglycones included isoquercitin (*m/z* 465.1021), quercetin (*m/z* 303.0495), galangin (*m/z* 271.0597), pinocembrin (*m/z* 257.0805), datiscetin (*m/z* 287.0545), cirsiliol (*m/z* 331.0807), and daidzein (*m/z* 255.0648), while the abundant glycosylated flavonoids included the datiscetin-derivative datiscin (*m/z* 595.1652) and two putative derivatives of kaempferol (*m/z* 449.1072 and *m/z* 639.1916) (**Figure 3**). The relative abundance of these flavonoids in *D. glomerata* roots and nodules, determined by *t*-tests, was not significantly different between roots and nodules.

In *M. sativa* nodules, the highly abundant flavonoids were exclusively isoflavonoids, including formononetin (*m/z* 269.0813), the most abundant, and its derivative the second most abundant flavonoid medicarpin (*m/z* 271.0968), as well as coumestrol (*m/z* 269.0449) and the genistein-derivative prunetin (*m/z* 285.0762) (**Figure 4**). Due to sample variability, statistically significant fold changes were not obtained for the majority of the identified *M. sativa* flavonoids between nodules and roots.

### Differential Accumulation of Flavonoids in *D. glomerata* Roots and Nodules

#### Flavonoids

In *D. glomerata* nodules, naringenin and pinocembrin (*m/z* 257.0805), both of which are flavanones from which other flavonoid classes are derived, were significantly increased over roots (**Figure 3**). Dihydrokaempferol (*m/z* 271.0597), an aglycone derivative of naringenin that is an intermediate in the synthesis of flavonols from naringenin, was also significantly increased in the nodule.

#### Flavonols and Flavones

As noted above, the most abundant flavonoids in *D. glomerata* nodules were the flavonols datiscetin and datiscin, as well as galangin, all of which are derivatives of pinocembrin (**Figure 3**). Other flavonols, including kaempferol and quercetin, were found to have several known and putative derivatives significantly more abundant in the nodules than roots as well. Three putative kaempferol glycosides were highly abundant in *D. glomerata* nodules: astragalin (*m/z* 449.1071), kaempferol 7-*O*-glucoside (*m/z* 449.1072), and demethoxycentaureidin 7-*O*-rutinoside (*m/z* 639.1916); and quercetin and its derivative isoquercetin (*m/z* 465.1021) were highly abundant in *D. glomerata* nodules.

No flavones, putative derivatives of apigenin and luteolin, were found to be significantly different between *D. glomerata* roots and nodules (**Figure 3**).

#### Isoflavonoids

Daidzein (*m/z* 255.0648) was one of the most abundant flavonoids in *D. glomerata* nodules and one of its derivatives, 6′-*O*-acetyldaidzin (*m/z* 459.1279) was significantly increased in nodules over roots (**Figure 3**). Additionally, genistein (*m/z* 271.0597), belonging to a separate isoflavonoid pathway, was also significantly more abundant in nodules than roots.

#### Anthocyanins

Only one anthocyanin was annotated from *D. glomerata*: peonidin 3-*O*-rutinoside (*m/z* 463.1229) and it was neither abundant nor significantly different in the nodule (**Figure 2**).

### Rutinose Glycosides Accumulated in *D. glomerata* Nodules

A considerable number of rutinose glycosides were identified in the flavonoids of *D. glomerata* roots and nodules, across several of the flavonoid classes, including datiscin (*m/z* 595.1652), one of the most abundant flavonoids identified (**Figure 3**). Other rutinose glycosides detected included rutin (quercetin-3-*O*-rutinoside, *m/z* 611.1600), narirutin (naringenin-7-*O*-rutinoside, *m/z* 419.1331), peonidin-3-*O*-rutinoside (*m/z* 463.1229), demethoxycentaureidin-7-*O*-rutinoside (*m/z* 639.1916), and kaempferol-3-*O*-rutinoside (*m/z* 595.1653). None of these were significantly different between roots and nodules, e.g., more abundant in nodules than roots. Of the rutinose glycosides, only narirutin was identified in *M. sativa* roots or nodules (**Figure 3**).

### Nodulation Enhanced Flavonoids in Inoculated *D. glomerata* Roots and Nodules

**FIGURE 5:**
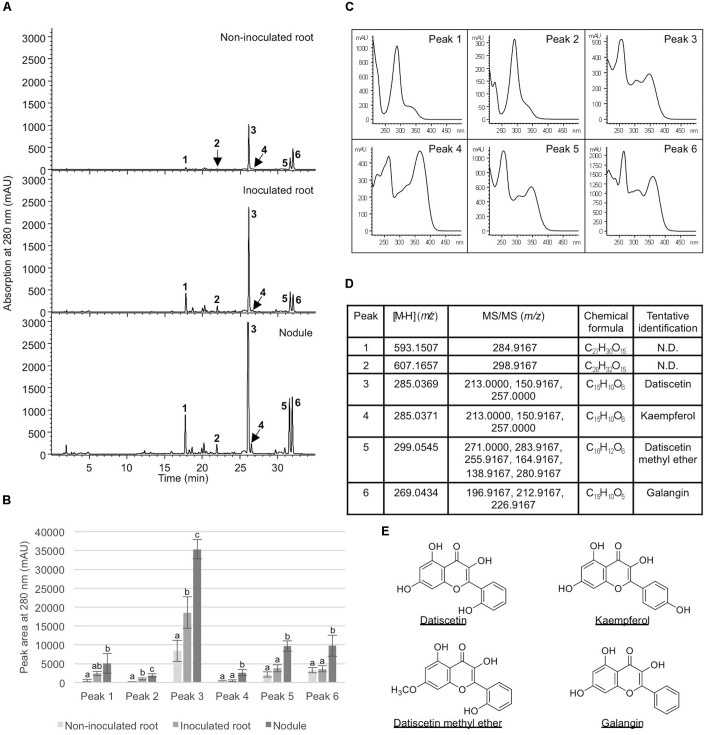
Analysis of metabolites in *Datisca glomerata* roots and nodules. **(A)** Representative HPLC elution profiles of phenolic metabolites extracted from non-inoculated and inoculated *D. glomerata* roots as well as nodules. Peaks (1–6) that show differential accumulation in roots and nodules are indicated. **(B)** Peak areas of differentially accumulated phenolic metabolites. Data shown are the average of four biological replicates ± standard deviation. Different letters indicate significant (*P* < 0.05) differences in metabolite levels among non-inoculated root, inoculated root, and nodule for each peak. **(C)** Absorption spectra of peaks 1–6. **(D)** MS and MS/MS analyses of peaks 1–6. **(E)** Chemical structures of tentatively identified phenolic metabolites in *D. glomerata* roots and nodules.

To examine how nodulation may influence flavonoid metabolites, we compared abundant metabolite composition of non-inoculated roots, inoculated roots, and nodules. Six metabolites (peaks 1–6) showed significantly greater accumulation in nodules in comparison to non-inoculated roots (**Figure 5**). Five (peaks 2–6) showed significantly greater accumulation in nodules than in the inoculated roots (**Figure 5**). In addition, two of the metabolites examined (peaks 2 and 3) showed significantly greater accumulation in inoculated roots when compared to non-inoculated roots. These metabolites were tentatively identified as structurally related flavonoids and flavonoid glycosides based on their retention times, UV absorption spectra, as well as MS and MS/MS data. The most abundant compound (peak 3) in all samples was tentatively identified as datiscetin (3,5,7,2′-Tetrahydroxyflavone) with other compounds (peaks 1, 2, and 5) potentially representing methylated or glycosylated derivatives. Peaks 4 and 6 were tentatively identified as kaempferol and galangin, respectively. The accumulation of these metabolites in roots and nodules are consistent with previous reported flavonoid profiles of *D. glomerata* (Bohm, 1988). These metabolite identifications were further supported by the observations that the retention time and UV absorption spectra for peak 3 (datiscetin; [M-H]^-^ *m/z* 285.0369) was inconsistent with an authentic standard of luteolin (*m/z* 286.2390) that has the same MS fragmentation pattern as datiscetin. Similarly, the retention time and UV absorption spectra for peak 6 (galangin; [M-H]^-^ *m/z* 269.0434) were inconsistent with authentic standards of apigenin (*m/z* 270.2400) and genistein (*m/z* 270.2400) that share the same MS fragmentation pattern as galangin. The data for peak 4 was consistent with an authentic standard of kaempferol analyzed in parallel.

### Expression of Phenylpropanoid Pathway Genes in Transcriptomes of *D. glomerata* and *M. truncatula* Roots and Nodules

**FIGURE 6:**
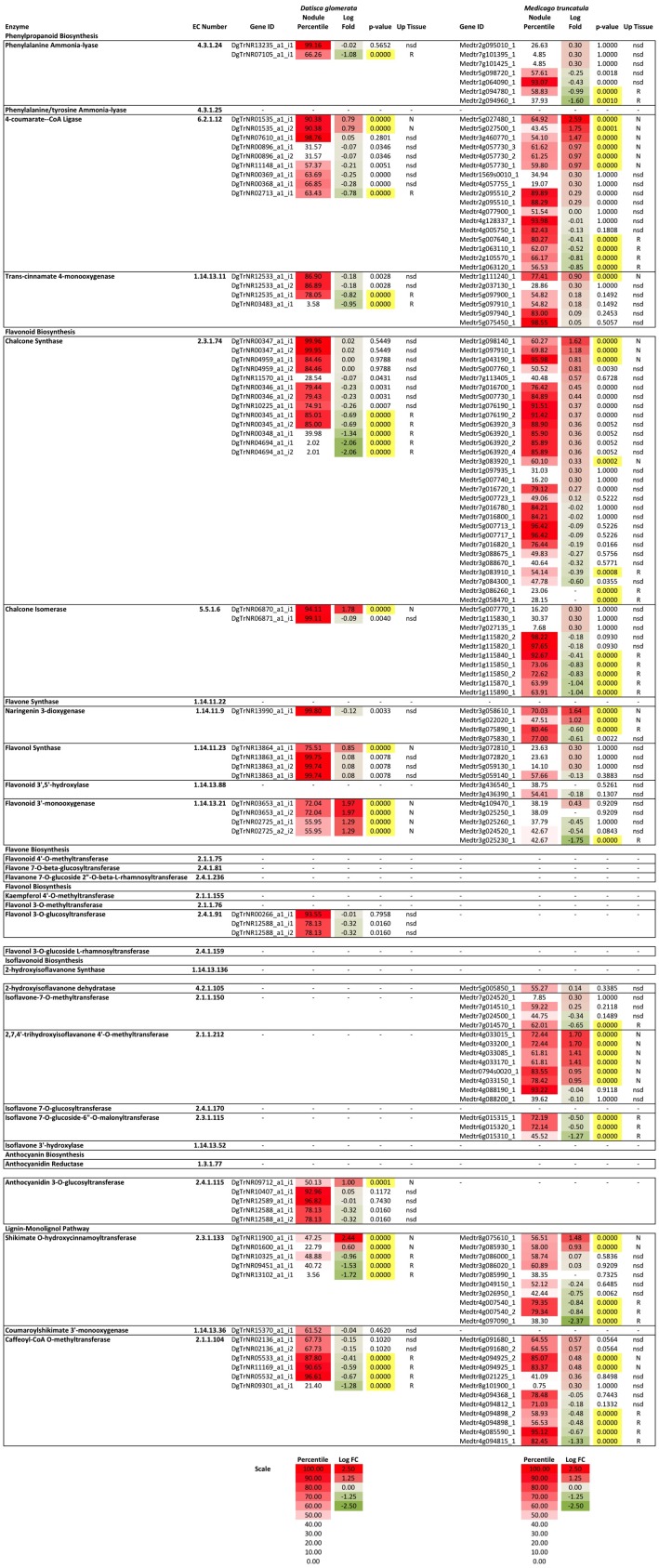
Heat map of flavonoid biosynthesis gene expression in *D. glomerata* and *M. truncatula* including relative expression in the nodule transcriptome based on percentile of all genes in their respective transcriptome (Percentile) and fold change between nodules and roots (Log fold change). Significance level used for differences in expression between roots and nodules was *p* < 0.001. Up-regulated tissues: N, nodules; R, roots; and nsd, no significant difference.

To understand the expression of the phenylpropanoid and flavonoid biosynthetic genes in roots and nodules, transcriptome data from roots and nodules of *D. glomerata* (Battenberg et al., 2018) and *M. truncatula* (Roux et al., 2014) were analyzed by differential expression between tissues and by comparing relative expression levels (as percentiles) of transcripts within each transcriptome. The reactions of the phenylpropanoid pathway, including the flavonoid branch, and the lignin-monolignol branch, are summarized in **Figure 1** and the relative expression of the genes comprising each branch are depicted in **Figure 6**. Early steps in the phenylpropanoid pathway preceding the flavonoid branch, e.g., phenylalanine ammonia-lyase (EC 4.3.1.24), and 4-coumarate-CoA ligase (4CL, EC 6.2.1.12) were highly expressed, above the 95th percentile in roots and nodules of both *D. glomerata* and *M. truncatula*, but were not significantly up-regulated or down-regulated in nodules of either host (**Figure 6**). The enzyme 4CL is responsible for the conversion of cinnamic acid to cinnamoyl-CoA and p-coumaric acid to p-coumaroyl-CoA, the precursor to flavonoid chalcones (**Figure 1**). In *D. glomerata* nodules, of the nine annotated 4CL transcripts, seven were not up-regulated relative to roots, while two (DgTrNR01535_a1_i1 and DgTrNR01535_a1_i2) were up-regulated over four-fold. In *M. truncatula* nodules, several annotated transcripts of this gene were also up-regulated between four- and six-fold, while one transcript was up-regulated greater than 300-fold (**Figure 6**).

Two of the four annotated transcripts of cinnamic acid 4-hydroxylase (C4H, EC 1.14.13.11), also known as *trans*-cinnamate 4-monooxygenase, were down-regulated in *D. glomerata* nodules almost 10-fold, while the other two were not statistically different (**Figure 6**) and none were expressed above the 87th percentile. C4H is the enzyme that catalyzes the last step in phenylpropanoid biosynthesis that precedes the separation of the naringenin flavonoid branch and the lignin-monolignol branch (**Figure 1**). In *M. truncatula*, by contrast, no C4H transcript was down-regulated in the nodule and one transcript (Medtr5g075450) was extremely highly expressed, above the 98th percentile of all genes in the transcriptome (**Figure 6**). The most highly expressed C4H transcripts expressed at significantly different levels in the transcriptomes of *D. glomerata* and *M. truncatula* nodules (*p* < 5 × 10^-4^).

As shown in **Figure 4**, in the flavonoid branch, one transcript of chalcone isomerase (EC 5.5.1.6), which catalyzes the synthesis of flavonoids from chalcones, was highly up-regulated in *D. glomerata* nodules (approximately 50-fold) while the second was not significantly different. Of the 10 chalcone isomerase transcripts in *M. truncatula*, on the other hand, none were up-regulated in the nodule, and half of them were significantly down-regulated. Transcripts annotated as encoding flavanone 3-dioxygenase (EC number 1.14.13.21), the enzyme responsible for the synthesis of both eriodictyol from naringenin and dihydroquercetin from dihydrokaempferol (precursors to flavones and flavanols, respectively), were also among the most up-regulated flavonoid biosynthesis genes identified in the *D. glomerata* nodule (approximately 20- and 100-fold). By contrast, *M. truncatula* showed down-regulation of these genes in the nodule or no significant difference between nodules and roots.

Four transcripts were annotated as flavonol synthase (flavonol biosynthesis, EC 1.14.11.23) in *D. glomerata*, one of which was significantly up-regulated in the nodule and was expressed in the 99th percentile in the nodule (**Figure 6**). In *M. truncatula* the corresponding transcripts were not significantly different between roots and nodules and were expressed in the 57th percentile at most, a marked contrast. Three flavonol-3-*O*-glucosyltransferases (EC 2.4.1.91) were annotated in *D. glomerata*; one of which showed high expression (above the 93rd percentile) with no significant change in expression between nodules and roots. In the transcriptome of *M. truncatula*, no transcripts were annotated as flavonol-3-*O*-glucosyltransferases.

Strikingly, no transcripts in the isoflavonoid pathway were annotated in the *D. glomerata* transcriptome, whereas, in the *M. truncatula* transcriptome, there were multiple transcripts annotated as encoding several enzymes in isoflavonoid biosynthesis (**Figure 6**). One of the eight transcripts of 2,7,4′-trihydroxyisoflavanone 4′-*O*-methyltransferase (EC 2.1.1.212) was highly expressed, above the 93rd percentile while four others were up-regulated over 10-fold in *M. truncatula* nodules. Enzymes that catalyze the synthesis of daidzein derivatives including formononetin and daidzein 7-*O*-glucoside were expressed but not significantly different between roots and nodules. Three transcripts of isoflavone 7-*O*-glucoside-6″-*O*-malonyltransferase (EC 2.3.1.115), an enzyme known to synthesize malonated daidzein derivatives, were annotated in *M. truncatula*. One was down-regulated over 10-fold in the *M. truncatula* nodule relative to the roots, falling to around the 45th percentile in the nodule while the other two remained around the 72nd percentile.

The gene encoding the earliest enzyme in anthocyanin biosynthesis, anthocyanidin 3-*O*-glucosyltransferase (EC 2.4.1.115), was up-regulated more than 10-fold in *D. glomerata* nodules and two other transcripts were expressed above the 90th percentile (**Figure 6**). By contrast, no anthocyanidin 3-*O*-glucosyltransferase transcripts were annotated in the nodule or root transcriptomes of *M. truncatula*.

Genes encoding the major enzymes in the lignin-monolignol branch of the phenylpropanoid pathway showed generally low expression in the transcriptome of *D. glomerata* (**Figure 6**). The most highly expressed transcript of the first enzyme in this branch, shikimate *O*-hydroxycinnamoyltransferase (HCT, EC 2.3.1.133) was expressed in the 48th percentile. Coumaroyl-shikimate 3′-monooxygenase (EC 1.14.13.36), the next gene in the pathway, was expressed in the 61st percentile. Caffeoyl CoA-*O*-methyltransferase (EC 2.1.104), converting caffeoyl-CoA to feruloyl-CoA, was the exception. Two transcripts were expressed in the 90th percentile in *D. glomerata* nodules, although transcripts were expressed up to 10-fold higher in the roots. In *M. truncatula* nodules, the transcripts encoding HCT were expressed much more highly than HCT in *D. glomerata* (*p* < 5 × 10^-5^), around the 80th percentile. No transcripts of coumaroyl-shikimate 3′-monooxygenase were annotated in *M. truncatula*.

## Discussion

### Metabolite and Expression Analyses Provide New Insights Into Flavonoid Metabolism in *D. glomerata* Roots and Nodules

A range of flavonoids were synthesized in roots and nodules of *D. glomerata*, including flavones, flavonols, flavanones, anthocyanins, and isoflavonoids. A greater number of flavonoids were more abundant in the nodule relative to roots than vice versa (**Figure 3**), suggesting an overall increase in flavonoid biosynthesis in the nodule.

For several highly-abundant metabolites analyzed, there was a general trend of increasing concentration, when comparing uninoculated roots with either roots post-inoculation, or root nodules (**Figure 5**). This suggests that inoculation with *Frankia* induces some change in flavonoid metabolism in roots, either systemically in nodulated plants, or locally by association with *Frankia* in the rhizosphere. In a split-root experiment on *M. sativa*, initiation of nodulation by application of either the symbiont *Sinorhizobium meliloti* or its Nod factor to roots on one side of the plant led to increased amounts of daidzein on the uninoculated side (Catford et al., 2006), supporting an interpretation that flavonoid biosynthesis and distribution is under global regulation in RNS, similar to, or part of, autoregulation (Reid et al., 2011).

Derivatives of the pinocembrin-derived subclass of flavonoids, especially datiscetin and datiscin, represented some of the most abundant molecules in *D. glomerata* nodules (**Figure 3**). The biosynthesis of datiscetin has been proposed to proceed by a reaction similar to the synthesis of galangin from pinobanksin through the addition of a 2C–3C double bond to dihydrodatiscetin that is itself synthesized from pinobanksin (Grambow and Grisebach, 1971). Galangin biosynthesis utilizes flavonol synthase (EC 1.14.11.23), which was found to be up-regulated four-fold in *D. glomerata* nodules (**Figure 6**). Because flavonol synthase has been shown to catalyze multiple reactions including both kaempferol and galangin biosynthesis (Miyahisa et al., 2006), it seems likely that datiscetin biosynthesis could be performed by this enzyme as well. Dihydrodatiscetin itself, however, was not identified in roots or nodules of *D. glomerata* in this study (**Figure 3**).

The prevalence of pinocembrin and its derivatives in *D. glomerata* is likely directly related to the relatively low expression of C4H (EC 1.14.13.11) (**Figure 5**), which appears to be a pivotal enzyme in the phenylpropanoid pathway in *D. glomerata* whose expression impacts two separate branch points (**Figure 1**): first, the enzyme catalyzes the conversion of the pinocembrin-precursor cinnamic acid to naringenin-precursor p-coumaric acid, and thus controls the balance between the pinocembrin and naringenin flavonoid branches. Because the early enzymes in the flavonoid branch, including chalcone synthase (EC 2.3.1.74) and naringenin 3-diooxygenase (EC 1.14.11.9) are multi-functional, catalyzing reactions with multiple substrates (Martens et al., 2010), the altered flux favoring cinnamoyl-CoA in *D. glomerata* likely directs the flow of flavonoid biosynthesis more toward pinocembrin and ultimately datiscetin and datiscin. Second, because expression of C4H is diminished relative to *M. truncatula*, the synthesis of naringenin-based flavonoids is likely aided in *D. glomerata* by lower expression of HCT (EC 2.3.1.133), which decreases metabolic flux to lignins in favor of flavonoid biosynthesis, a pattern similar to what was shown to occur when HCT was down-regulated in *M. sativa* by Gallego-Giraldo et al. (2014).

C4H has been shown to be the major rate-limiting step in lignin biosynthesis (Anterola and Lewis, 2002) suggesting that it also functions to control the relative flux of phenylpropanoids between flavonoid and lignin biosynthesis. Control of flux between flavonoid and lignin branches of the phenylpropanoid pathway in plants could be useful for crop improvement to enhance flavonoid content, particularly the pinocembrin pathway. Flavonoids in general are nutritional antioxidants, and pinocembrin specifically has shown both antitumor and neuroprotective capabilities (Rasul et al., 2013).

In addition to datiscetin, a flavonol synthesized from pinocembrin as discussed above, other flavonols were abundant in *D. glomerata* roots and nodules, particularly quercetin and its glycosides (**Figure 3**). Flavonol synthase (EC 1.14.11.23) was very highly expressed in *D. glomerata* roots and nodules, with one transcript expressed above the 99th percentile, compared to 57th percentile at the highest in *M. truncatula* nodules; and the other transcript was significantly up-regulated in the *D. glomerata* nodule seven-fold (**Figure 6**), reflecting the great abundance of flavonols, which may perform a variety of roles in nodules. Quercetin has been shown to regulate auxin gradients in roots during nodule formation as measured by a *gusA* gene-auxin response promoter construct (Mathesius et al., 1998). Quercetin showed a higher level of auxin transport inhibiting activity than kaempferol, apigenin, naringenin, or genistein; glycosylation decreased the auxin-inhibitory effect (Mathesius et al., 1998, 2015). Interestingly, in the laser-capture microdissection study of developmental gene expression in *M. truncatula* performed by Roux et al. (2014), 100% of flavonol synthase transcripts (Medtr5g059140) in nodules were found in zone FIID, the second distal fraction of the nodule. Cells in this zone are undergoing expansion and rhizobial infection (Roux et al., 2014), making it the zone where auxin gradient inhibition is likely required during the nodulation process (Mathesius et al., 2015). Additionally, quercetin has been shown to arrest cell division and cause DNA breaks leading to endoreduplication in eukaryotic cells *in vitro* (Cantero et al., 2006). This may suggest another potential role for flavonols in the formation of symbiotic cells in nodules (Vinardell et al., 2003), in addition to CCS52A-mediated endoreduplication, as described by Adachi et al. (2011). Finally, flavonols have been shown to protect enzymes from deactivation by nitric oxide and peroxynitrite (Heijnen et al., 2001). Nitric oxide is a signaling molecule required for nodule formation, however, it also deactivates enzymes important for symbiosis, including nitrogenase and glutamine synthetase, by nitration of tyrosine residues (Melo et al., 2011). Flavonoids in general have been found to protect enzymes by scavenging nitric oxide and peroxynitrite. Heijnen et al. (2001), found that galangin had strong scavenging activity and correlated this activity with the hydroxyl group on the third carbon; this would suggest that datiscetin is a strong peroxynitrite scavenger as well.

A feature of the flavonoid glycosides in *D. glomerata* roots and nodules was the frequent occurrence of rutinose glycosides. Six flavonoids were identified with rutinose glycosylations. Rutinose is a common glycosylation of flavonoids in many plants (Cuyckens et al., 2001), and was previously shown to occur in leaves of *Datisca cannabina* (Bohm, 1988). Rutinose was reported to occur as an exceptionally abundant free sugar in roots, nodules, and leaves of *D. glomerata* and *D. cannabina* (Schubert et al., 2010) that could not be hydrolyzed in cell extracts. Thus it is hypothesized that rutinose is synthesized as a flavonoid glycosylation and then released as a free sugar (Schubert et al., 2010).

### Potential Nod Gene Inducing Flavonoids in *Datisca glomerata*

In legume symbioses a range of flavonoids, predominantly aglycones, have been shown to induce the expression of *nod* genes in rhizobia, including flavones (luteolin), flavanones (eriodictyol and naringenin), and chalcones in *Medicago* and isoflavonoids (daidzein and genistein) in *Glycine* (Phillips and Tsai, 1992). In this study all of these metabolites were identified in roots and nodules of both *D. glomerata* and *M. sativa*, with the exception of chalcones, which were not detected in either host (**Figures 3**, **4**). The identified molecules could play potentially similar roles in the *D. glomerata* symbiosis as in *Medicago*. *D. glomerata* is nodulated by Cluster 2 *Frankia* strains whose genomes have been shown to contain *nodABC* gene homologs that are expressed in symbiosis (Persson et al., 2015; Nguyen et al., 2016); however, it is unknown whether flavonoids are involved in the regulation of these genes since no clear *nodD* homolog has been identified (Persson et al., 2015). Our results indicate that molecules in the pinocembrin pathway, including galangin and datiscetin, are possible candidates for a similar role in *D. glomerata*, because they are synthesized in great abundance (**Figure 3**) and are synthesized by uninoculated roots as well as inoculated roots and nodules (**Figure 5**) suggesting they are present in roots before the roots are infected.

Pinocembrin, the precursor to galangin and datiscetin, has been reported in several actinorhizal hosts. In the Fagales, it was found in the leaves and flowers of members of the genus *Alnus* (Betulaceae; Ren et al., 2017), as well as in leaves of *Myrica* and *Comptonia* (Myricaceae), all hosts nodulated by *Frankia* belonging to Cluster 1 (Wollenweber et al., 1985; Normand et al., 1996). However, flavonoids from leaf exudates of eight species of *Ceanothus* (Rhamnaceae, in Rosales), another genus that, like *D. glomerata*, is nodulated by Cluster 2 *Frankia* (Normand et al., 1996), were not reported to include pinocembrin or its derivatives (Wollenweber et al., 2004).

### *D. glomerata* and *M. sativa* Share Similar Classes of Flavonoids but Differ in Abundance

Both *D. glomerata* and *M. sativa* metabolomes contained flavonoids in a range of classes. Both hosts contained similar classes of flavonoids, including flavanones, flavonols, flavonoid glycosides, isoflavonoids, and isoflavonoid glycosides, but within each class they varied significantly in diversity (**Figure 2**). In both hosts the abundance of the majority of flavonoids was not statistically different between their roots and nodules.

The largest differences between the two plants were in the amount within particular flavonoid classes produced, most notably, in the pinocembrin pathway as discussed above, and in the isoflavonoids. All four of the highly abundant flavonoids in *M. sativa* nodules were isoflavonoids (**Figure 4**). This conforms to earlier reports highlighting isoflavonoids as most abundant in nodules of *M. sativa* (Tiller et al., 1994) and *M. truncatula* (Modolo et al., 2007; Staszkow et al., 2011). In *D. glomerata*, however, only one of the 10 highly abundant flavonoids (daidzein) was an isoflavonoid (**Figure 3**). Isoflavonoids identified in *D. glomerata* were found early in the biosynthetic pathway (**Figure 3**) whereas *M. sativa* included highly abundant derived isoflavonoids, including the pterocarpin medicarpin and its precursor formononetin (**Figure 4**). Pterocarpins are primarily found in legumes (Dewick, 1982) and have been shown to function as antimicrobials that show much greater inhibition of gram-positive bacteria than gram-negatives (Gnanamanickam and Smith, 1980). However, Auguy et al. (2011) reported expression of isoflavone reductase in the actinorhizal plant *C. glauca*, suggesting the synthesis of the more derived isoflavonoids similar to the *Medicago*. This leaves the distribution and role of isoflavonoids in actinorhizal plants unresolved.

## Conclusion

We present the first comparison of metabolic profiles of flavonoids from both roots and nodules of two host plants within the NFC, *D. glomerata* and *M. sativa*, with transcriptomes obtained from roots and nodules, in the context of phenylpropanoid biosynthetic pathways. The most abundant flavonoids in *D. glomerata* were derivatives of pinocembrin as well as naringenin whereas flavonoids from *M. sativa* were isoflavonoids and derivatives of naringenin. These findings correlate with the pattern of expression of cinnamic acid 4-hydroxylase (C4H), in the transcriptomes of the two hosts. *D. glomerata* showed relatively low expression of C4H in nodules compared to *M. truncatula*, suggesting a role for this enzyme in directing the flow of the phenylpropanoid pathway between the pinocembrin branch and the naringenin branch. Similarly, shikimate *O*-hydroxycinnamoyltrasferase (HCT), the link between the flavonoid and monolignol branches of the phenylpropanoid pathway, also showed lower expression in *D. glomerata*, supporting a difference in metabolic flux between the two hosts that favors flavonoids over monolignol/lignin production in *D. glomerata*.

Flavonoids of the same classes were present in roots and nodules of both *D. glomerata* and *M. sativa*, including flavanones, flavonols, and isoflavonoids, suggesting similar roles for flavonoids during nodule development and symbiosis across lineages in the NFC. Common roles may include symbiotic signaling, protection of enzymes from nitration, nodule organogenesis including phytohormone regulation, and cell-cycle modification. To identify symbiotically important flavonoids, further higher resolution transcriptome studies including spatio-temporal sampling (as in Roux et al., 2014, or Larrainzar et al., 2015) in combination with metabolomics profiling are needed. Secondly, responses of *Frankia* in culture to purified flavonoids identified as unique or amplified in their respective hosts should be measured at the transcriptomic level, and in terms of nodulation patterns, to evaluate a broader role for flavonoids as signaling molecules in the actinorhizal symbioses.

## Data Availability Statements

Metabolome data from *D. glomerata* and *M. sativa* generated in this study are presented in **Supplementary Table S2**. Transcriptome data for *D. glomerata* and *M. truncatula* used were obtained from Roux et al. (2014) and Battenberg et al. (2018), respectively.

## Author Contributions

IG developed metabolomic and transcriptomic profiles, determined patterns of flavonoid metabolism, and substantially wrote the manuscript; each author contributed to the writing of the manuscript for their, respective, section. In addition, KB provided annotated transcriptomes and was responsible for the plant material. AV developed analytical methods for and performed metabolomic analyses. AW performed LC-MS (MS/MS) analyses on peaks identified by HPLC and analyzed glycosyltransferase transcriptome data. LT carried out HPLC analyses and provided data interpretation. OF contributed to metabolomics project design and manuscript editing. AB provided project oversight and contributed to manuscript construction.

## Conflict of Interest Statement

The authors declare that the research was conducted in the absence of any commercial or financial relationships that could be construed as a potential conflict of interest.

## Supplementary Material

FIGURE S1 Head-to-tail comparisons of MS/MS spectra of the most abundant flavonoids identified in *D. glomerata.* Reference library spectra are shown in red, experimental spectra are given in blue. Metadata include the similarity dot product and reverse dot product scores, InChIKey, and the structure of annotated compounds.

FIGURE S2 Head-to-tail comparisons of MS/MS spectra of the most abundant flavonoids identified in *M. sativa*. Reference library spectra are shown in red, experimental spectra are given in blue. Metadata include the similarity dot product and reverse dot product scores, InChIKey, and the structure of annotated compounds.

TABLE S1 Collection data for root and nodule samples of *D. glomerata* and *M. sativa*.

TABLE S2 Flavonoid metabolome data obtained from LCMS analyses of *D. glomerata* and *M. sativa* nodules and roots.

TABLE S3 Number of flavonoids annotated in each class in *D. glomerata* and *M. sativa* roots and nodules and chi-square test comparing the distribution of classes by proportion of total flavonoids in each host.
