# Supplementary material for: Distinctive Patterns of Flavonoid Biosynthesis in Roots and Nodules of Datisca glomerata and Medicago spp. Revealed by Metabolomic and Gene Expression Profiles
Source: Front Plant Sci. 2018 Oct 10;9:1463. doi: 10.3389/fpls.2018.01463 (PMC6192435; doi:10.3389/fpls.2018.01463)

## Slide 1
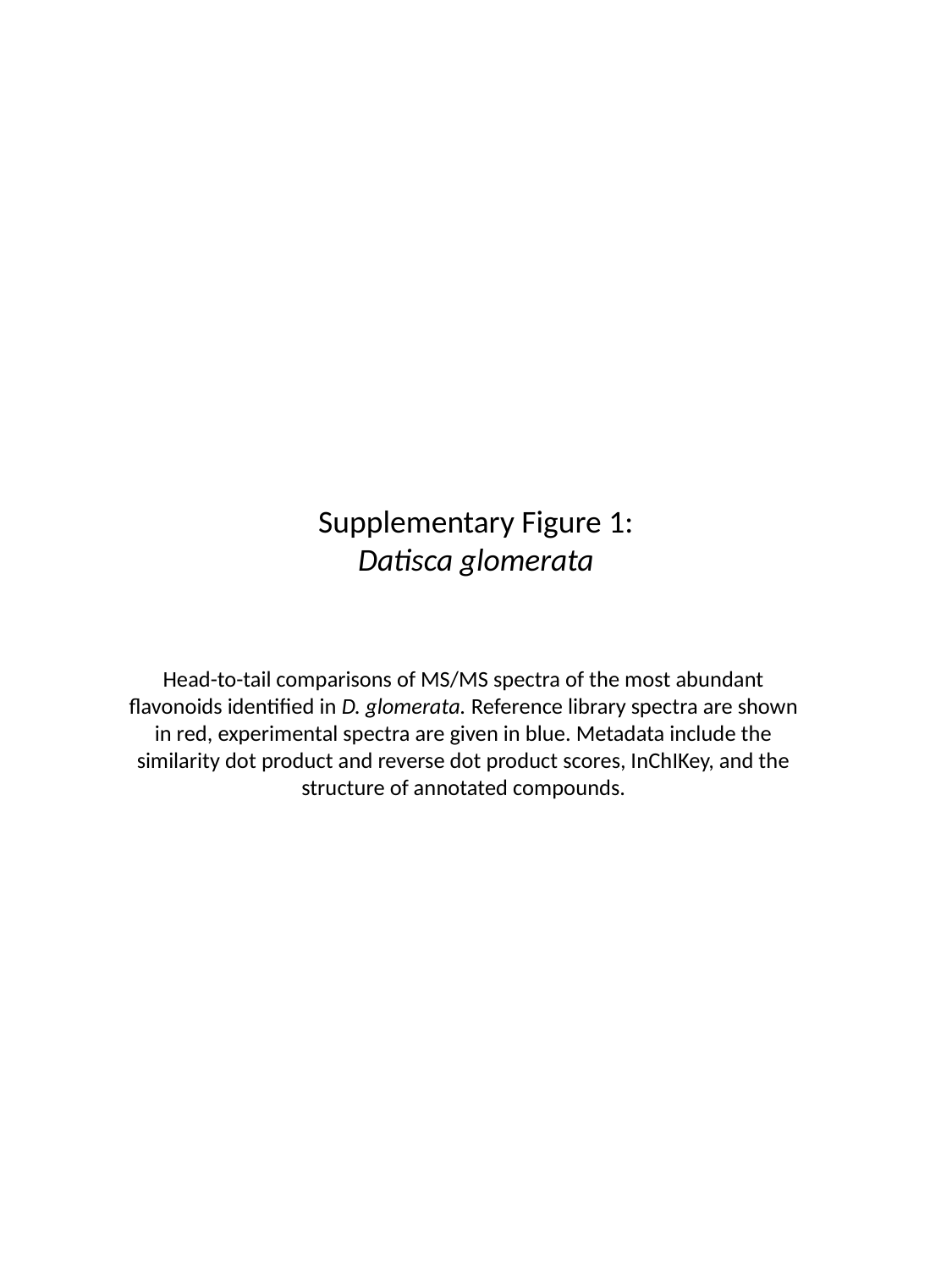

# Supplementary Figure 1: Datisca glomerata
Head-to-tail comparisons of MS/MS spectra of the most abundant flavonoids identified in D. glomerata. Reference library spectra are shown in red, experimental spectra are given in blue. Metadata include the similarity dot product and reverse dot product scores, InChIKey, and the structure of annotated compounds.

## Slide 2
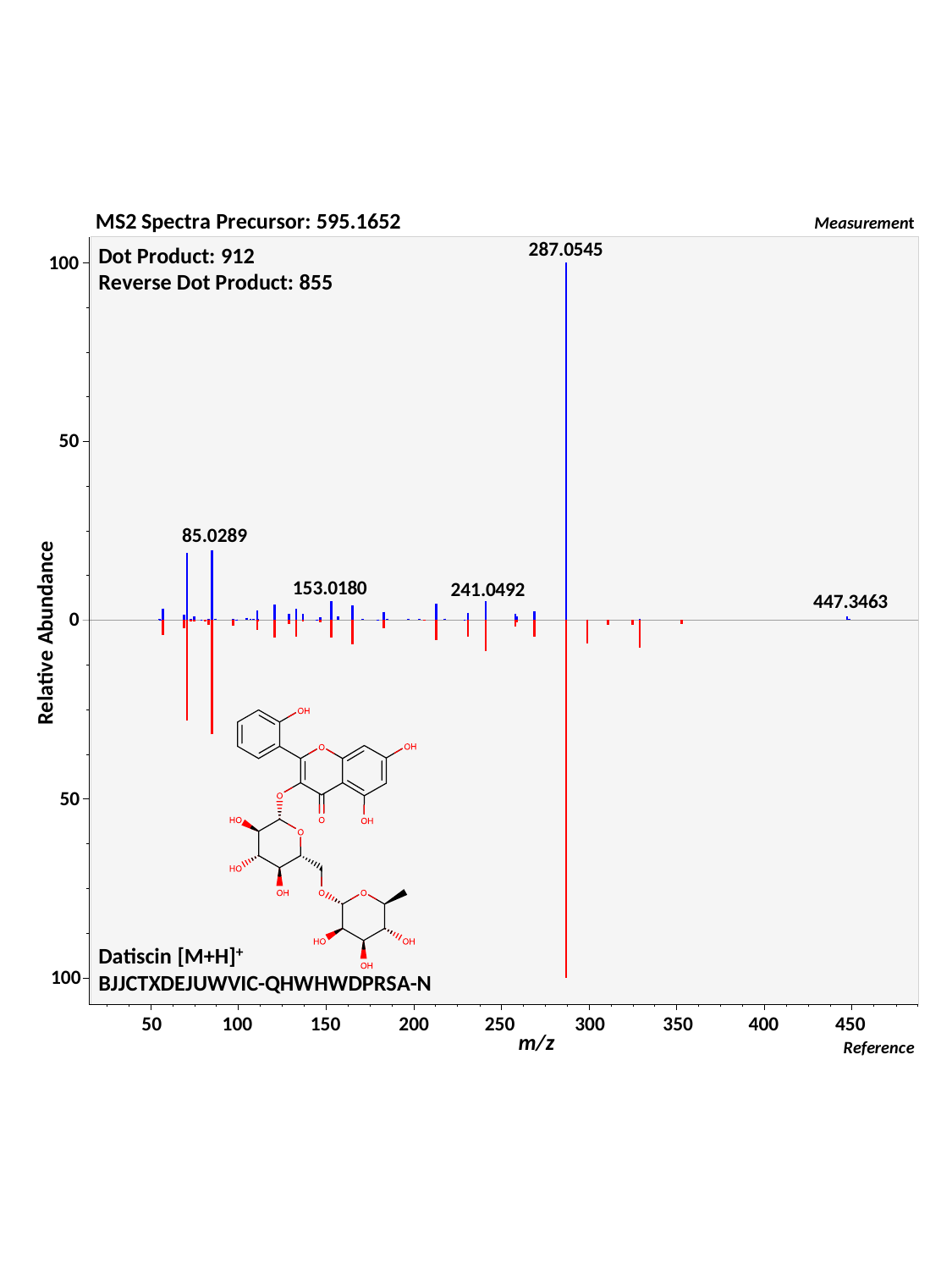

## Slide 3
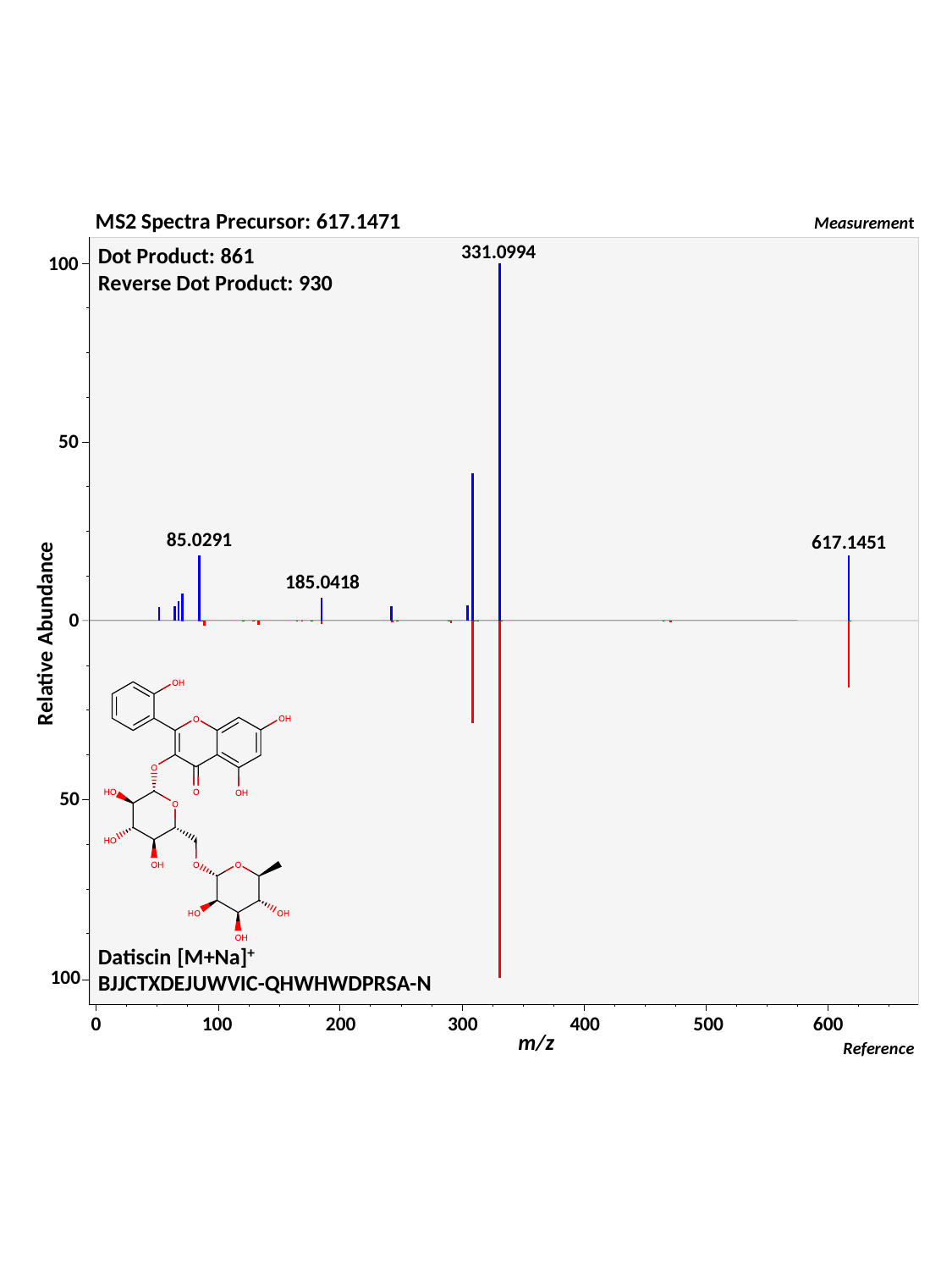

## Slide 4
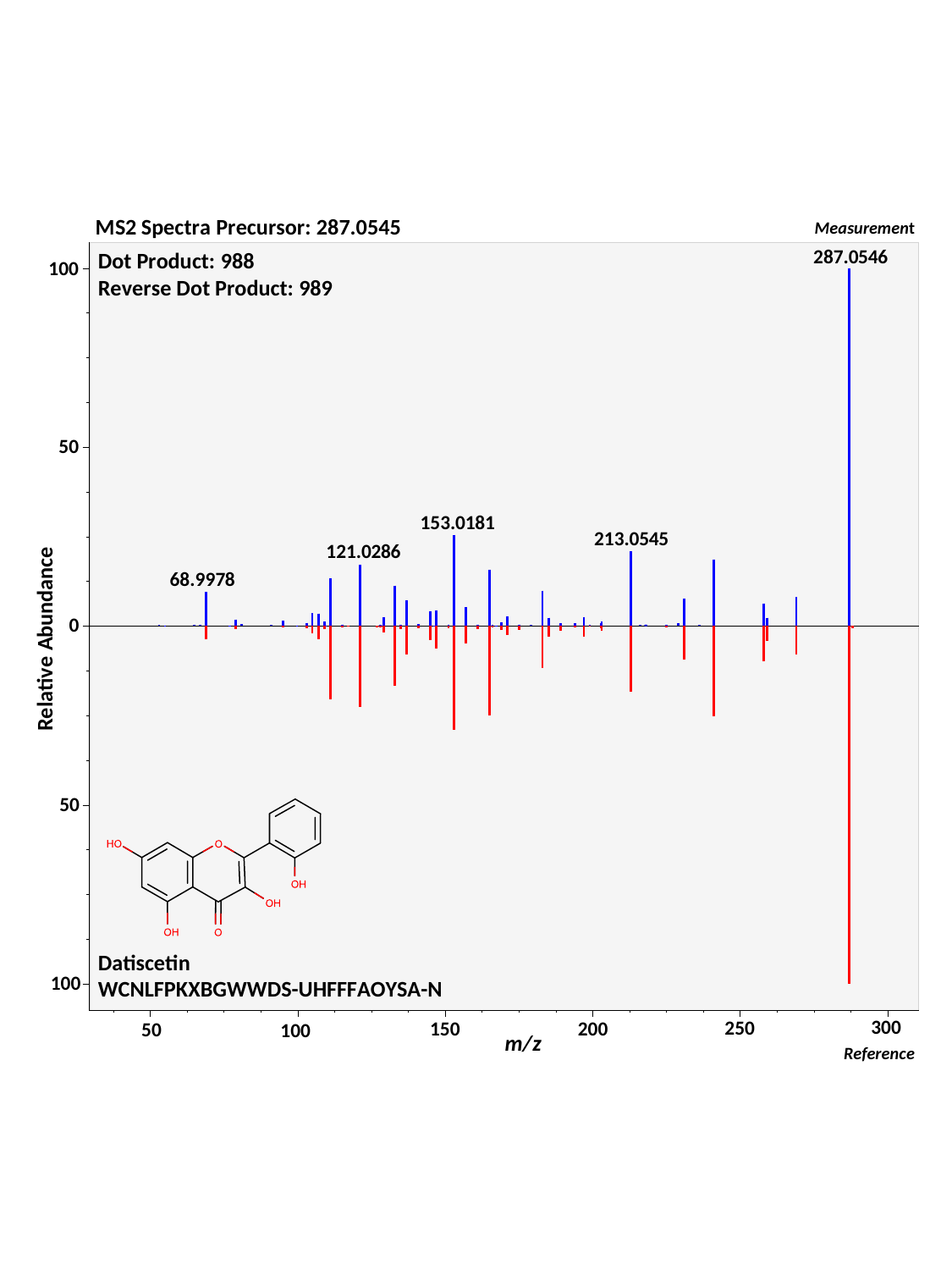

## Slide 5
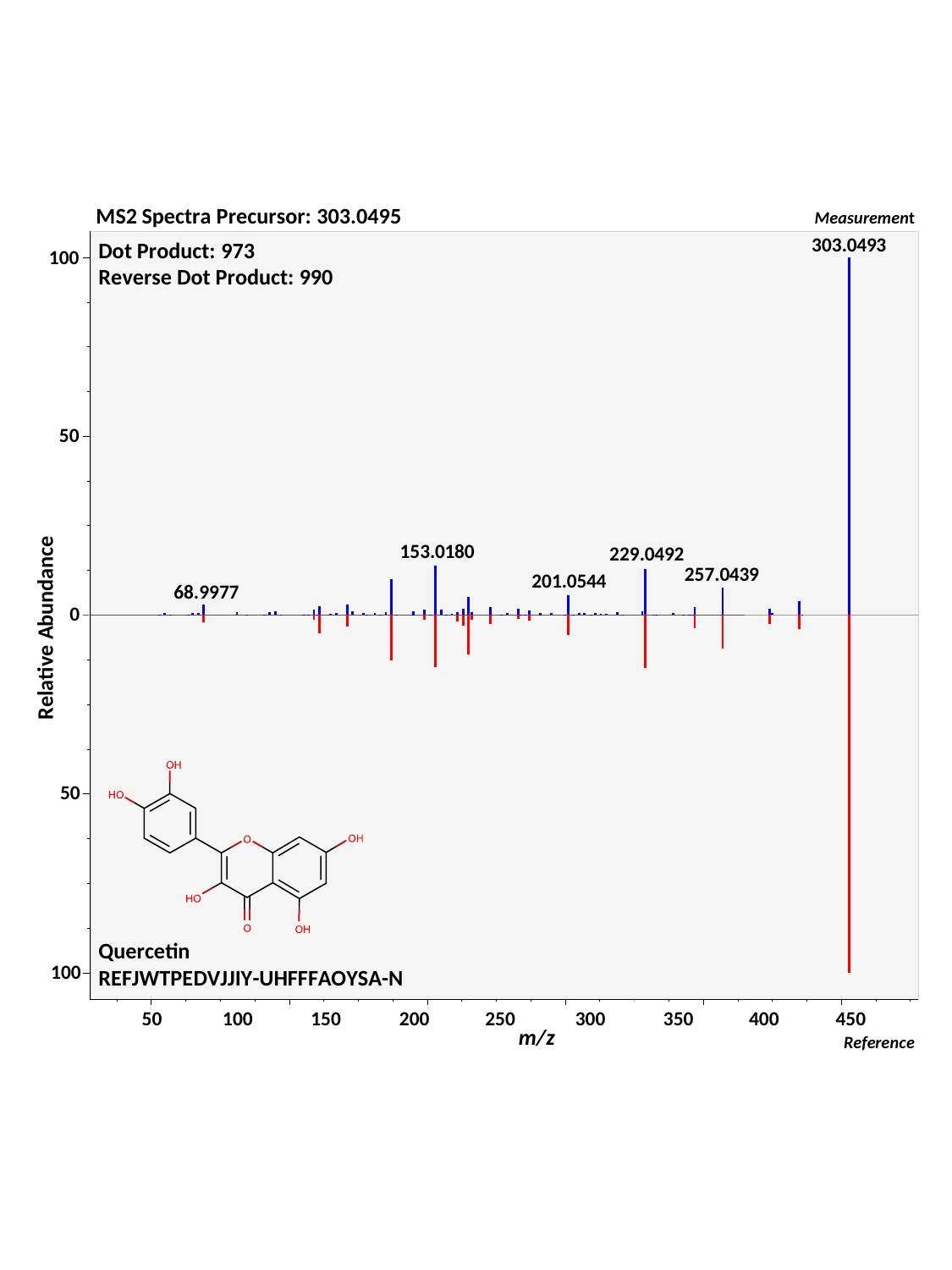

## Slide 6
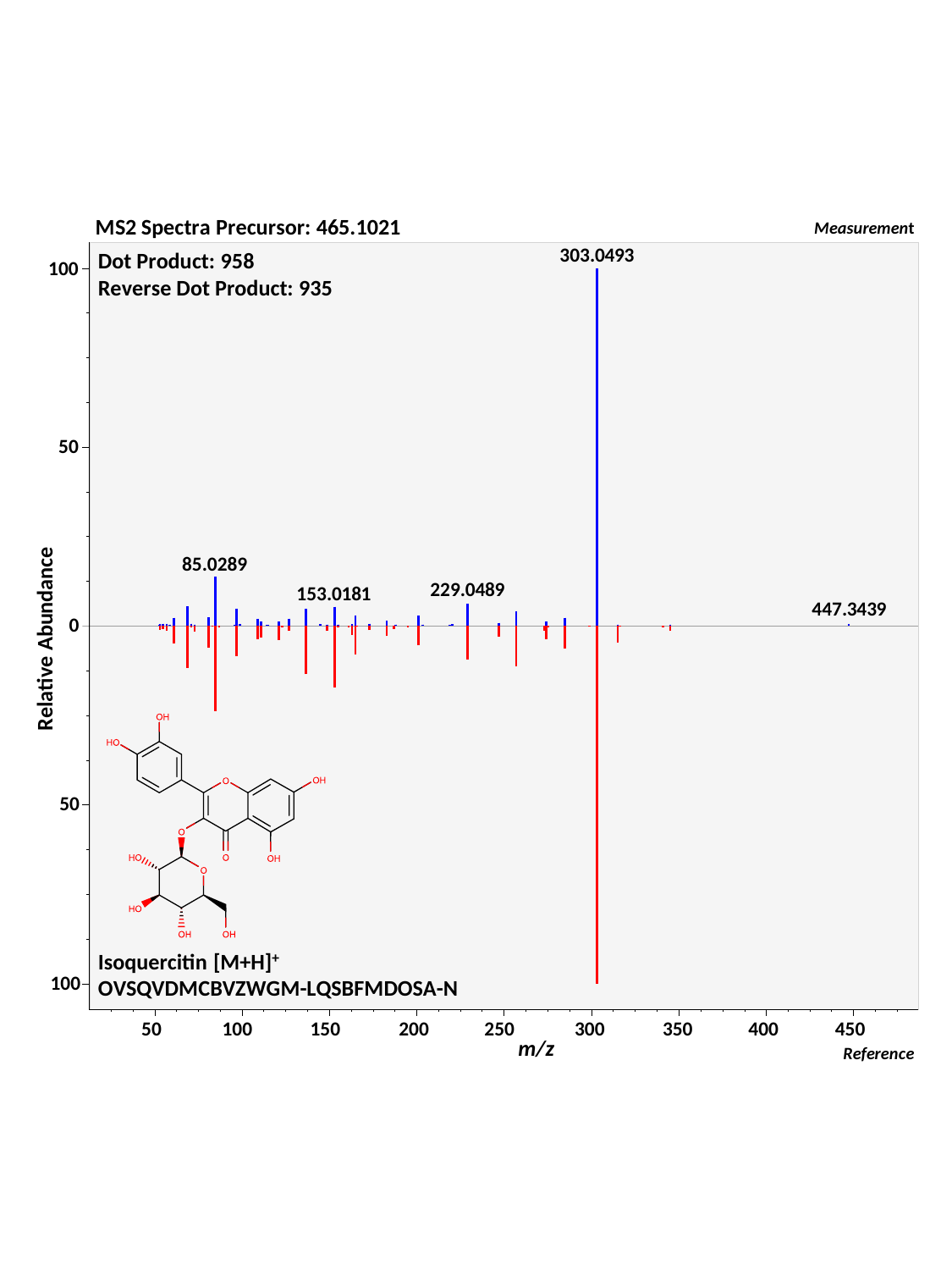

## Slide 7
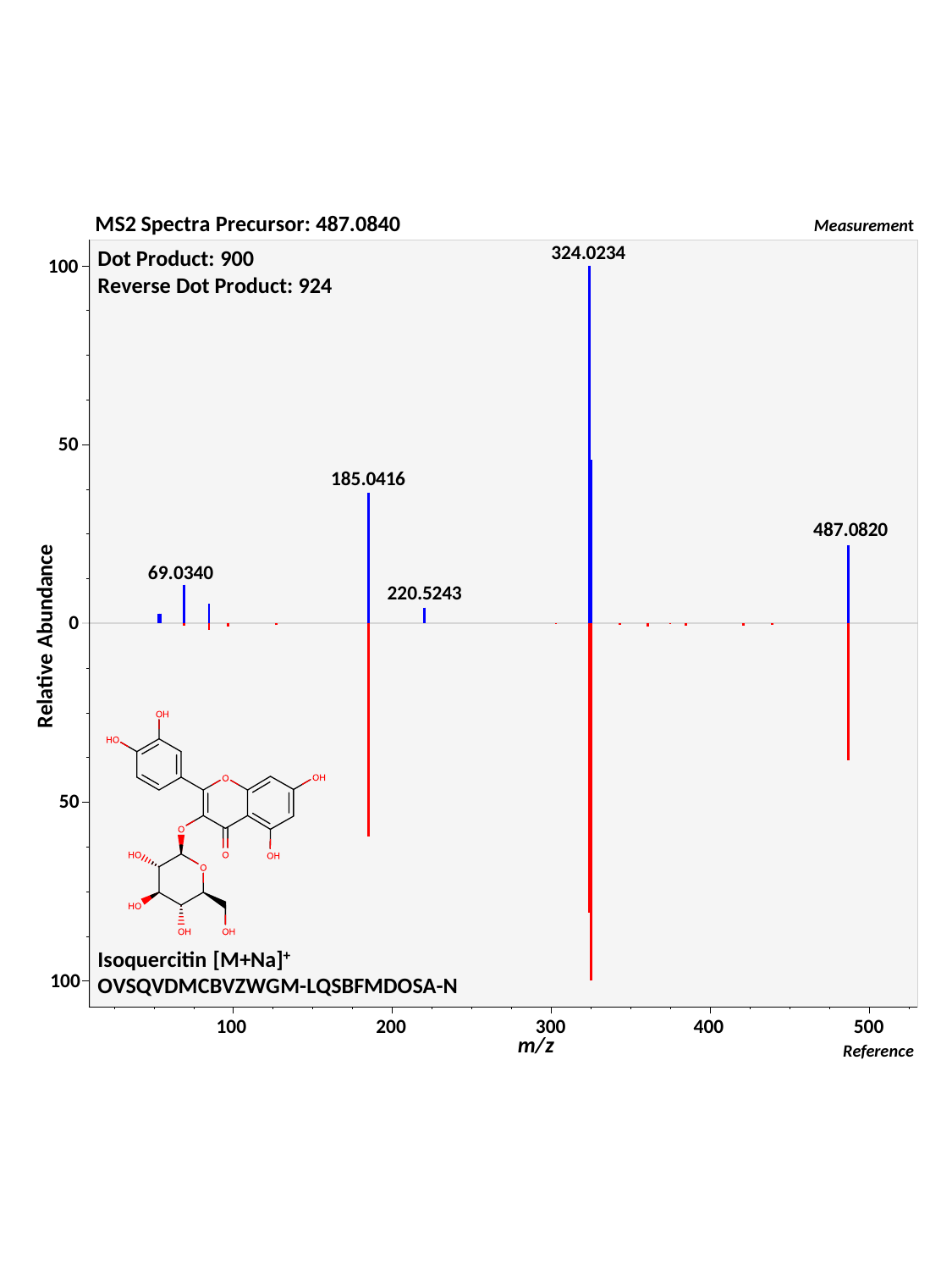

## Slide 8
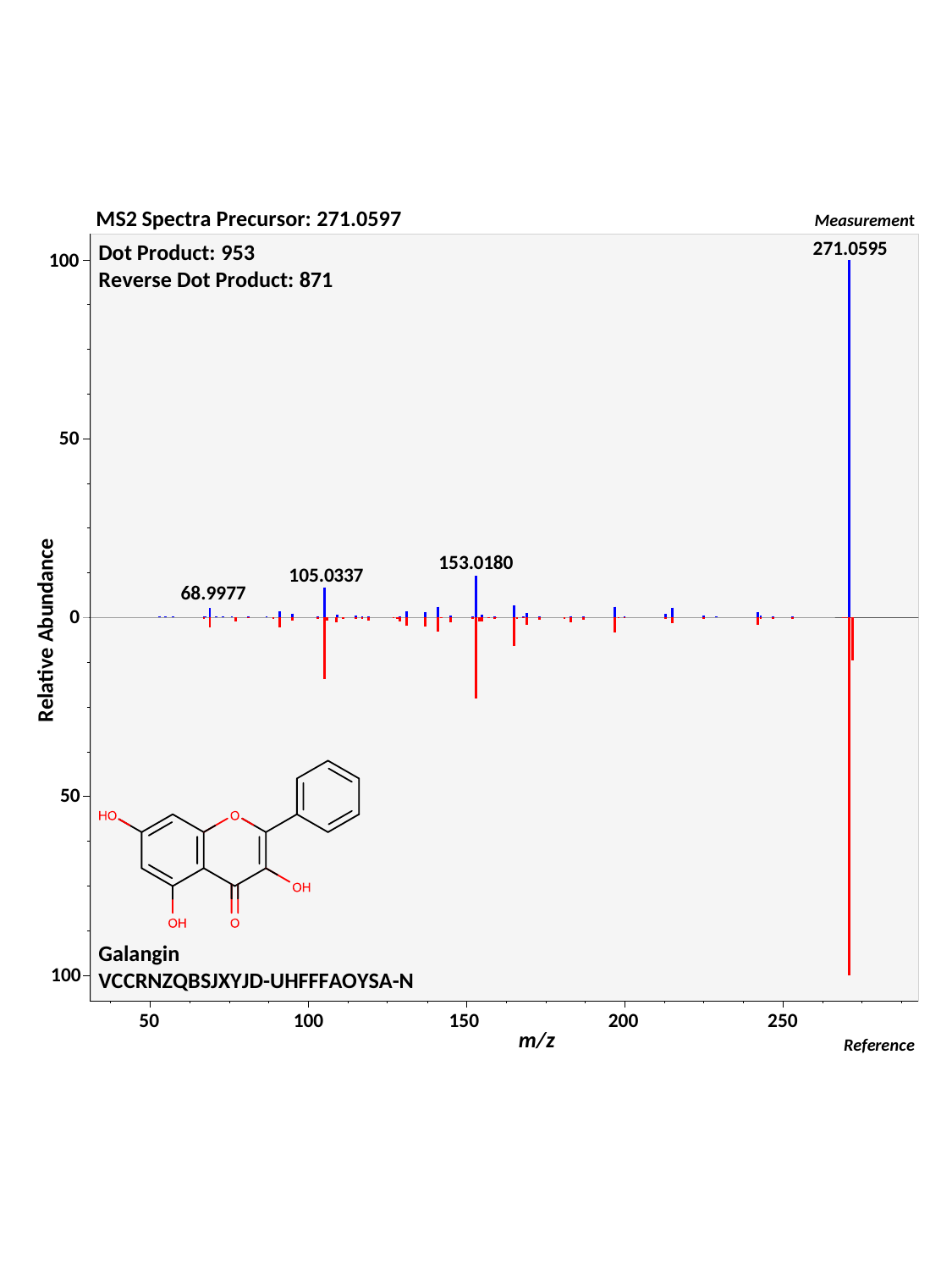

## Slide 9
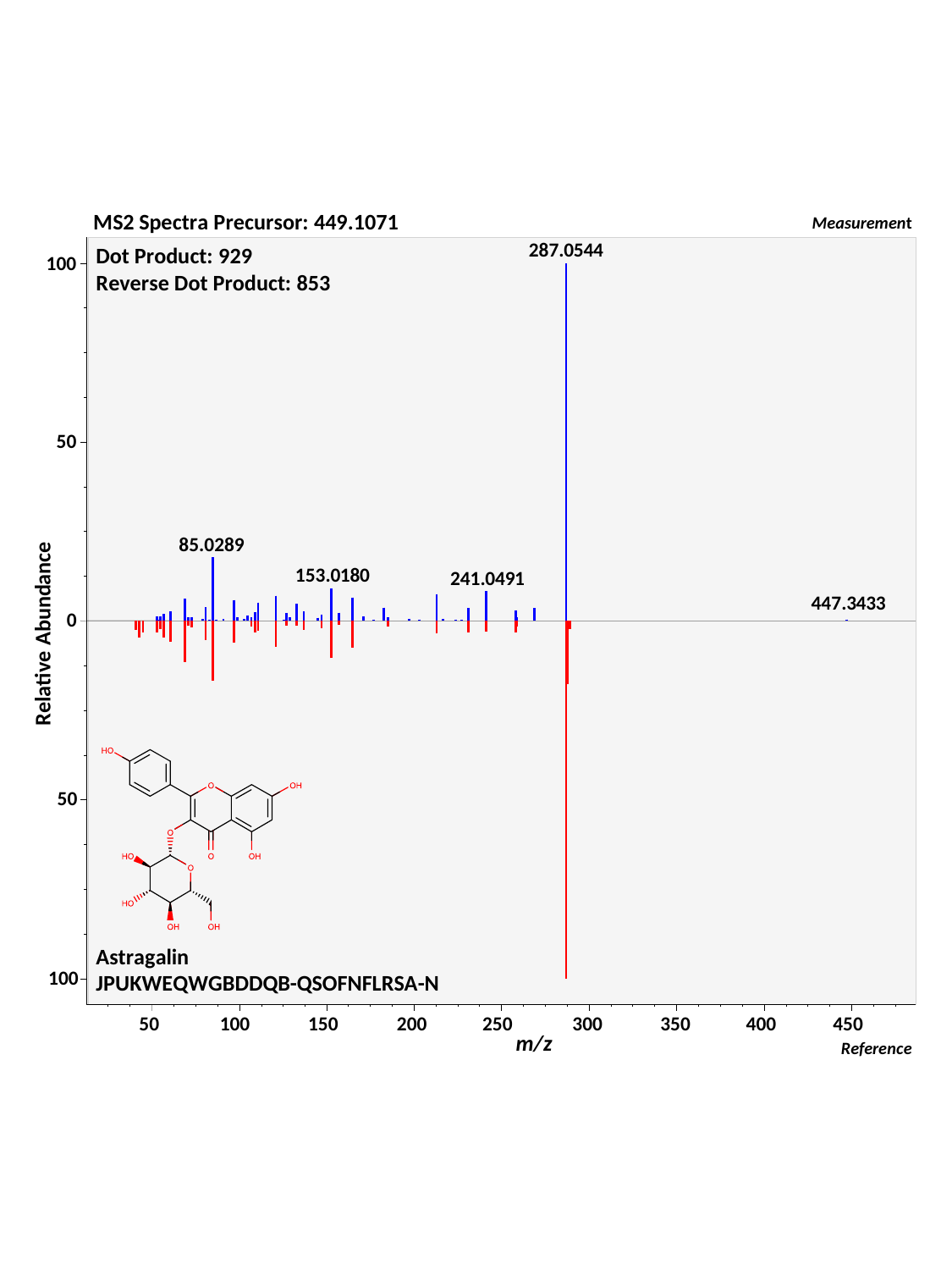

## Slide 10
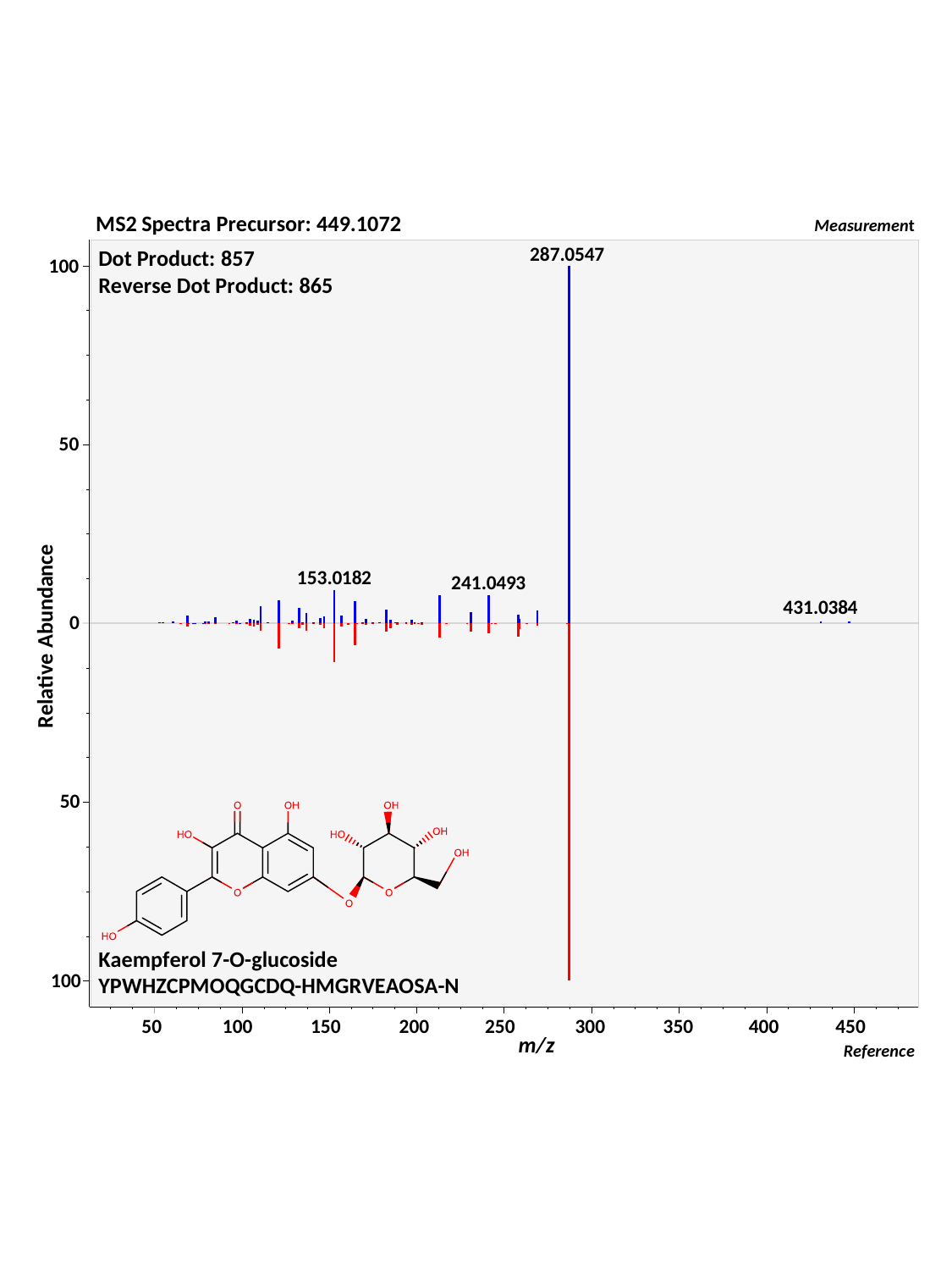

## Slide 11
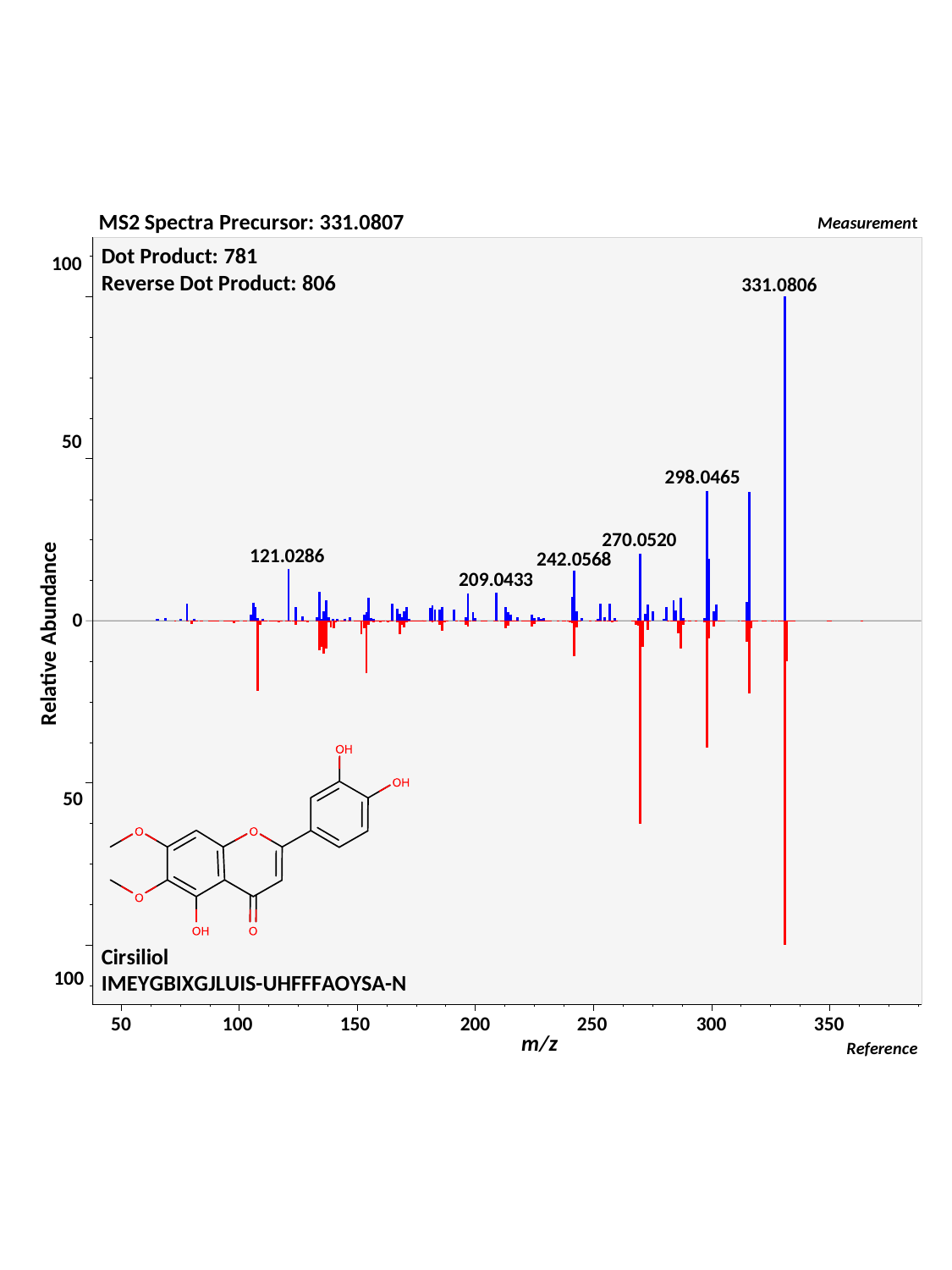

## Slide 12
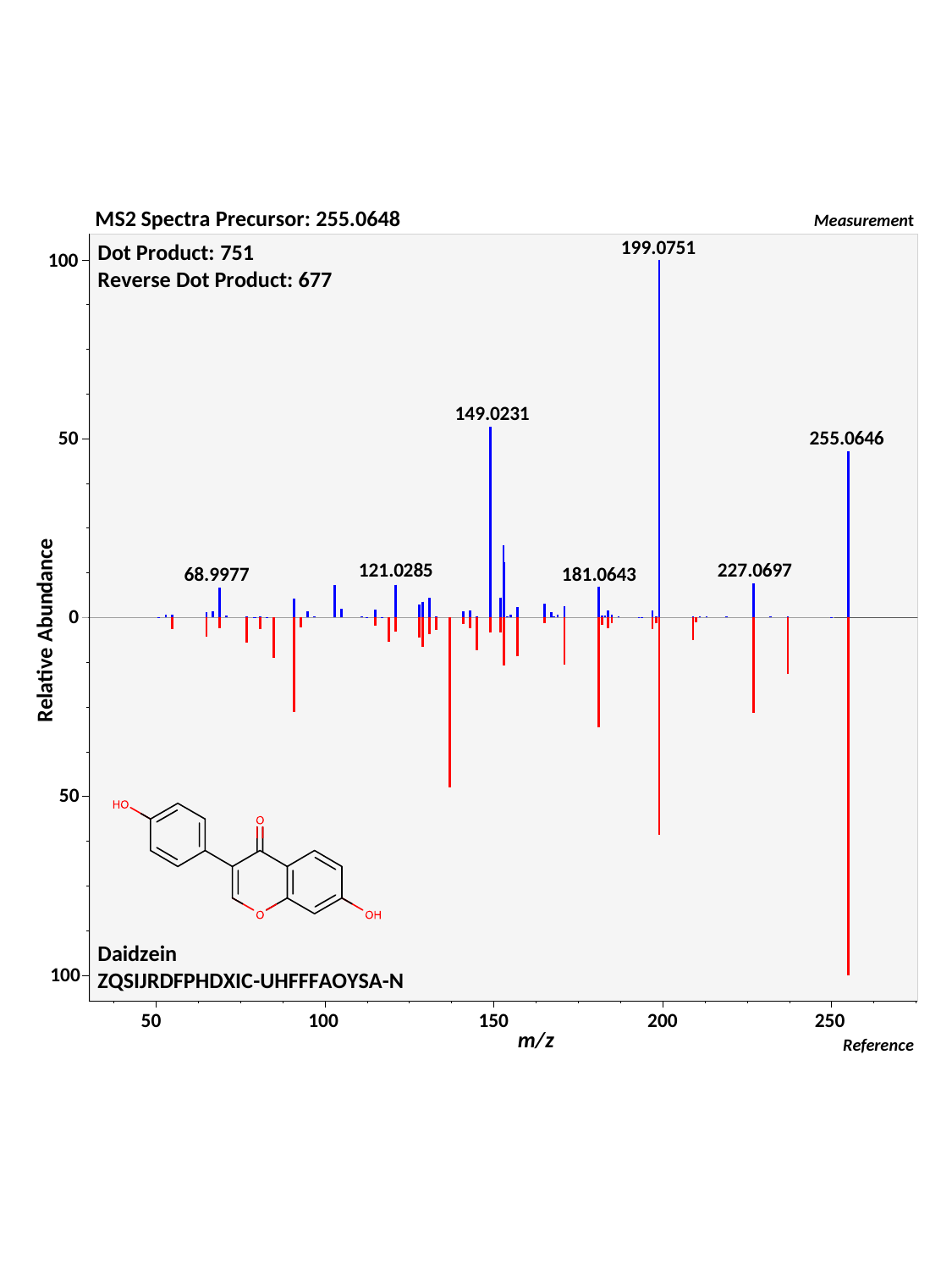

## Slide 13
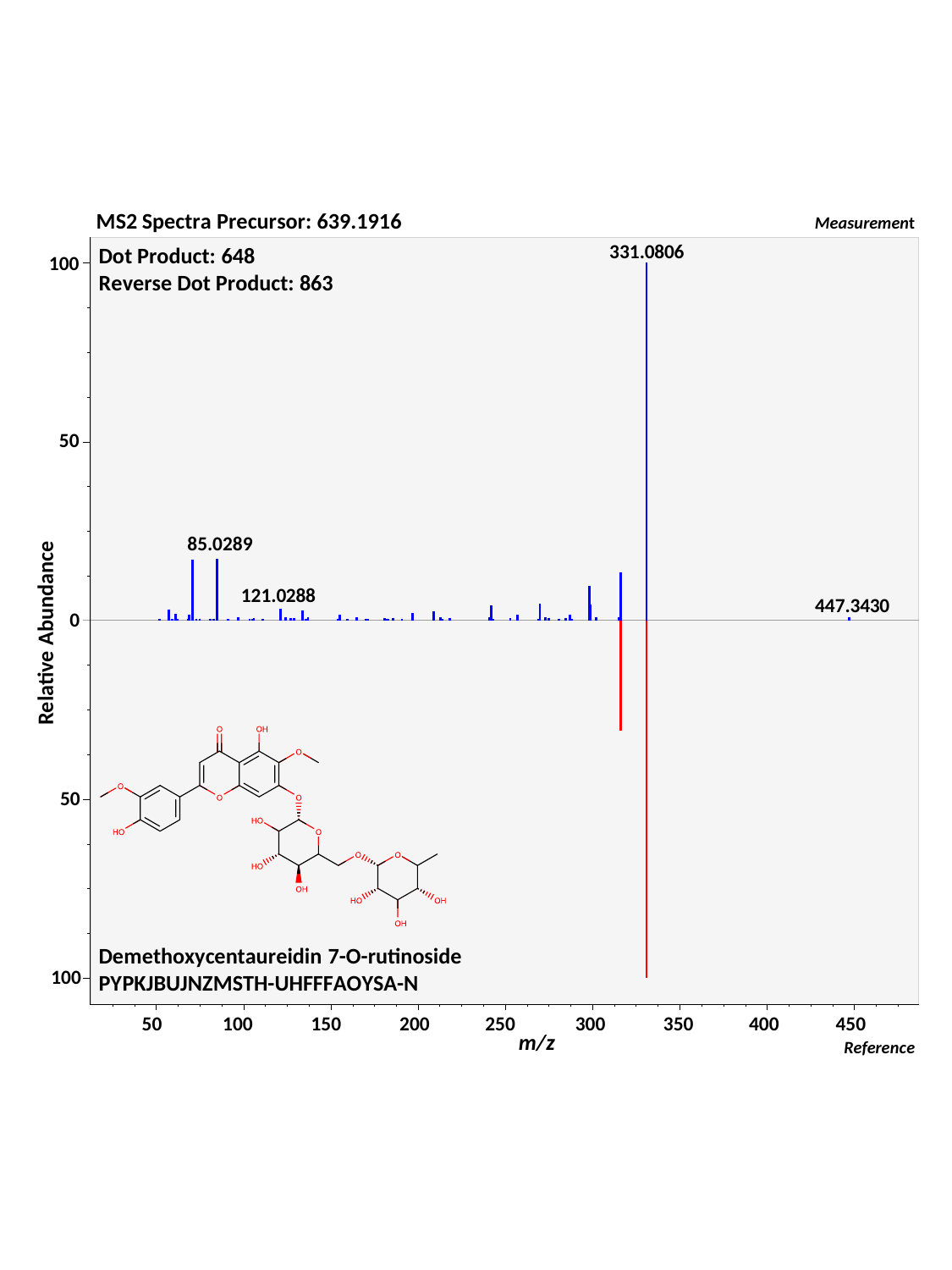

Supplement: FIGURE S1 — Head-to-tail comparisons of MS/MS spectra of the most abundant flavonoids identified in D. glomerata. Reference library spectra are shown in red, experimental spectra are given in blue. Metadata include the similarity dot product and reverse dot product scores, InChIKey, and the structure of annotated compounds. [file Presentation_1.PPTX]
